# Bayesian networks and structural equation models reveal genetic causal relationships between productivity, defense, and climate-adaptability traits in interior lodgepole pine

**DOI:** 10.1093/g3journal/jkaf308

**Published:** 2025-12-24

**Authors:** Eduardo P Cappa, Jennifer G Klutsch, Andy Benowicz, Sebastián Munilla, Shawn D Mansfield, Nadir Erbilgin, Barb R Thomas, Yousry A El-Kassaby

**Affiliations:** Instituto Nacional de Tecnología Agropecuaria (INTA), Instituto de Recursos Biológicos, Centro de Investigación en Recursos Naturales, De Los Reseros y Dr. Nicolás Repetto s/n, Hurlingham, Buenos Aires 1686, Argentina; Consejo Nacional de Investigaciones Científicas y Técnicas (CONICET), Buenos Aires, Argentina; Natural Resources Canada, Canadian Forest Service, Northern Forestry Centre, Edmonton, Alberta T6H 3S5, Canada; Department of Renewable Resources, University of Alberta, 442 Earth Sciences Bldg., Edmonton, Alberta T6G 2E3, Canada; Forest Stewardship and Trade Branch, Alberta Forestry and Parks, Edmonton, Alberta T6H 5T6, Canada; Departamento de Producción Animal, Facultad de Agronomía, Universidad de Buenos Aires, Ciudad Autónoma de Buenos Aires 1417, Argentina; CONICET – Universidad de Buenos Aires, Instituto de Investigaciones en Producción Animal (INPA), Buenos Aires, Argentina; Department of Wood Science, Faculty of Forestry, University of British Columbia, Vancouver, British Columbia V6T 1Z4, Canada; Department of Botany, Faculty of Science, University of British Columbia, Vancouver, British Columbia V6T 1Z4, Canada; Department of Renewable Resources, University of Alberta, 442 Earth Sciences Bldg., Edmonton, Alberta T6G 2E3, Canada; Department of Renewable Resources, University of Alberta, 442 Earth Sciences Bldg., Edmonton, Alberta T6G 2E3, Canada; Department of Forest and Conservation Sciences, Faculty of Forestry, University of British Columbia, Vancouver, British Columbia V6T 1Z4, Canada

**Keywords:** Bayesian networks, structural equation model, genetic causal effects, multiple-trait analysis, genetic parameters, lodgepole pine

## Abstract

This study investigates the integration of Bayesian networks (BN) and structural equation models (SEM) to explore genomic relationships among nine traits related to productivity, defense, and climate-adaptability in an interior lodgepole pine breeding program. Data from 392 open-pollinated trees, genotyped with 25,099 SNP markers, were analyzed. The traditional multitrait model (MTM) served as a benchmark for comparing SEM in estimating genetic (co)variance components, genetic correlations, breeding value (BV) predictions, and predictive ability, using both pedigree- (ABLUP) and genomic-based (GBLUP) individual-tree mixed models. The Hill-Climbing algorithm identified 12 significant causal structures (λ) among traits. Strong positive causal effects included tree height (HT) on wood density (WD) (λ_HT→WD_ = 0.413) and on stable carbon isotope ratio (C13) (λ_HT→C13_ = 0.565), and limonene (LIMO) on carbon assimilation rate (CAR) (λ_LIMO→CAR_ = 0.368). The most influential causal relationship was HT → C13, followed by resistance to western gall rust (WGR) → CAR, CAR → LIMO, and WGR → C13. SEM incorporated these relationships, capturing both direct and indirect effects. Compared with MTM, SEM yielded lower residual variances, higher additive variances, and higher heritability estimates for all traits. The λ values from SEM correlated strongly with genetic correlations (0.932), with similarly high correlations between models (0.929), though SEM produced lower posterior mean correlations. BV correlations between models were high (ABLUP > 0.82, GBLUP > 0.84), but some reranking occurred among the top 39 trees (ABLUP > 0.71, GBLUP > 0.42). ABLUP and GBLUP-SEM models outperformed MTM in predictive ability, with mean gains of 6.62 and 6.03%, mainly for conditioned traits. BN-SEM enhances understanding of trait networks, improving genomic evaluations and breeding strategies in forest trees.

## Introduction

Genetic evaluation programs have consistently expanded the number of traits they encompass over time. When evaluating multiple, interconnected traits, the multitrait model (MTM) pioneered by Henderson and Quaas (1976) has become a crucial tool. MTM enables the simultaneous estimation of breeding values (BVs) for each trait while accounting for genetic and environmental covariances among them. In tree breeding, specifically, the MTM has been particularly valuable for improving the accuracy of predicting BVs (Cappa et al. 2018). This feature is especially true for traits with low heritability or limited data, where joint modeling with one or more easily measured, inexpensive, genetically correlated, and highly heritable traits can substantially boost prediction accuracy (Jurcic et al. 2023).

A core principle of MTMs is that all relationships between traits are represented by symmetric, linear associations measured by covariances or correlations (Valente et al. 2010). These measures quantify how traits tend to vary together, but they do not necessarily imply a causal relationship. In reality, phenotypic traits can have direct causal effects on one another (Rosa et al. 2011). Traditional MTMs used in standard quantitative genetics do not explicitly address these functional (or causal) relationships between phenotypes.

To model these causal pathways, one must move beyond standard MTM to the framework of SEM (Valente et al. 2010). SEM allows researchers to represent and test specific hypotheses about the causal relationships between traits. This approach provides a powerful means to understand the intricate interplay of traits in a biological system and to move beyond simply observing correlated variation. As such, a SEM offers a more nuanced picture of trait interdependencies, which can lead to a better understanding of the underlying biology and potentially more effective breeding strategies.

Building on the fundamental work of Gianola and Sorensen (2004), which integrated SEM into the quantitative genetic framework, researchers have gained the ability to address linear feedback or recursive relationships among traits, relationships that standard MTMs cannot capture. This seminal work spurred the application of SEM across diverse fields, including animal breeding (see recent review by Varona and González-Recio (2023)), crop breeding and genetics (Töpner et al. 2017; He et al. 2021), adaptation to drought stress in forest trees (Sebastian-Azcona et al. 2025), and ecological studies (reviewed by Fan et al. (2016)). In animal breeding, for example, several studies have compared MTM and SEM in estimating model parameters (Bouwman et al. 2014; Inoue et al. 2016; Mokhtari et al. 2018; Oberpenning et al. 2023). These comparisons have further illuminated the potential of SEM to provide a more nuanced understanding of trait interdependencies. The review by Varona and González-Recio (2023) provides a detailed overview of SEM applications in this latter field. Authors foresee that SEM will see increased adoption in animal breeding due to its capacity for causal inference and its potential for implementation in real-world operational applications (Varona and González-Recio 2023). However, the comparative efficiency of SEM relative to MTMs in this context remains unknown in forest tree breeding, representing a key area of research and application.

A core assumption of SEM lies in the requirement that the connections between traits must be predefined to estimate their magnitude. Typically, these underlying causal structures are determined based on existing information or hypotheses. In this context, Bayesian network (BN) learning methods (Scutari and Denis 2021) provide a powerful alternative by providing an efficient means to uncover the underlying network structure in multivariate data. The BN methods can deduce relationships between correlated variables within the network (Töpner et al. 2017; Yu et al. 2019), essentially learning the causal structure from the data. This ability provides a critical preliminary step before implementing SEM, such that the learned BN structure informs the model structure for SEM, allowing for a data-driven approach to specifying causal pathways.

To date, the integration of BN with SEM remains unexplored in forest tree breeding. Here, we demonstrate its application using data on nine productivity, defense, and climate-adaptability traits from an interior lodgepole pine (*Pinus contorta* subsp. *latifolia* Douglas) breeding population. The research had three primary objectives: 1) to employ a BN approach to identify potential causal structures among productivity, defense, and climate-adaptability traits in lodgepole pine; 2) to use the BN-inferred structure to fit an SEM and to quantify the relationships between the aforementioned traits; and 3) to compare the performance of MTM and SEM in terms of estimated genetic (co)variance components, genetic correlations for all trait combinations, BV prediction, and predictive ability using both pedigree-based (ABLUP) and genomic-based (GBLUP) individual-tree mixed models.

## Materials and methods

### Genetic material and trial description

This study employed data from an open-pollinated (OP) lodgepole pine progeny test located near Timeau, Alberta, Canada (54°41′ N 115°18′ W). The progeny test site is part of the Region C breeding program (FGRMS, 2016) owned and managed by West Fraser - Blue Ridge Lumber, Inc. at the time of the study. The entire breeding population of 224 OP families was selected from five natural stands (provenances) within Region C that included: Deer Mountain, Inverness River, Judy Creek, Swan Hills, and Virginia Hills (Dhir 1983). The experimental design was a “sets-in-reps” design with five replications (blocks). Each replication contained 21 sets, and within each set, trees were planted in 4-tree family row plots at a 2.5 m × 2.5 m spacing (John and Sadoway 2019). Further details about this test site can be found in Cappa et al. (2022a, 2022c).

### Phenotypic data

This study analyzed nine key traits of importance to the lodgepole pine breeding program, which typically requires extensive phenotyping efforts. These traits were grouped into three categories: productivity, defense, and climate adaptability. The productivity traits included total tree height (HT), measured at age 30, and wood density (WD), measured from 5 mm increment cores taken at breast height (1.3 m). Whole-tree WD was calculated as a weighted average of individual tree ring densities, accounting for ring width and using oven-dry weight.

The defense traits included, in the first place, pest resistance, which was assessed based on infection severity to western gall rust (WGR), caused by *Endocronartium harknessii* (J.P. Moore) Y. Hirats, evaluated at age 36 using a four-category resistance rating, derived from a seven-category scoring system (Cappa et al. 2022c). The WGR rating was subsequently transformed into a continuous normal score following the approach of Gianola and Norton (1981). In addition, two defense-related monoterpene compounds were also measured: concentrations of limonene (LIMO) and total monoterpenes (T_MONO), quantified from phloem tissue collected from each tree (Cappa et al. 2022a).

The four climate adaptability traits were: 1) drought resistance (RES), which quantifies the reduction in incremental growth during a drought event (Cappa et al. 2022b); 2) growth decline index (DECL), reflecting long-term growth reductions due to recurring droughts, derived from tree-ring data, and calculated by comparing growth in the final 5 yr to peak growth (1997–2001); 3) stable carbon isotope ratio (C13), measured using increment cores (Cappa et al. 2022b); and 4) carbon assimilation rate (CAR), calculated as the ratio of the photosynthetic rate per unit leaf area (A_area_) to the leaf mass per unit area (LMA), yielding a mass-based photosynthetic rate (A_mass_) that reflects carbon assimilation efficiency per unit leaf biomass (Wei et al. 2022). Except for RES and CAR, all phenotypes were previously studied in Cappa et al. (2022a, 2022c).

These nine traits were chosen for exploration using BN and for fitting SEM to quantify their interrelationships. Prior to BN analyses and model fitting, several preprocessing steps were applied to the phenotypic data. To improve data normality, a logarithmic transformation was used to DECL and the monoterpene compounds LIMO and T_MONO. All phenotypic data were spatially adjusted to account for the experimental design effects. Design-adjusted phenotype data were obtained for each tree and trait by subtracting the estimated replication and set-within-replication effects from the original phenotype values (Cappa et al. 2022a, 2022c). Finally, all trait data were standardized to have a mean of zero and a variance of one. Table 1 provides a comprehensive list of the traits, the number of trees with data for each trait, and descriptive statistics for each phenotypic trait in its original scale (*i.e.* without design adjustment and standardization).

**Table 1.** Overview of the descriptive statistics for the Timeau interior lodgepole pine dataset, including trait, number (No.) of records, mean, standard deviation (SD), minimum (Min.), and maximum (Max.) values. See text for trait abbreviations.

Trait	No. of records	Mean	SD	Min.	Max.
HT	392	1,051.92	102.61	610.00	1,350.00
WGR	392	1.86	1.06	1	4
WD	367	402.08	28.07	335.85	556.21
RES	368	0.76	0.19	0.16	1.42
DECL	328	2.00	1.00	0.66	11.10
C13	392	−26.06	0.54	−27.53	−24.27
LIMO	391	223.71	360.93	7.01	2,878.31
T_MONO	391	4,374.70	2,581.77	170.88	16,998.76
CAR	378	577.34	204.49	54.16	1,416.40

### Genomic data

Genotypes from a genotyping-by-sequencing (GBS) platform (Chen et al. 2013), as described by Cappa et al. (2022c), were available for 402 trees. After filtering the SNP dataset for a 30% data proportion and a minor allele frequency ≥1%, a final set of 392 trees and 25,099 (25 K) SNPs was retained. Missing data were imputed using the mean allele frequency (Ratcliffe et al. 2015). The SNPs were generated using the reference-free UNEAK pipeline (Lu et al. 2013), as no lodgepole pine genome reference assembly was available.

After pedigree correction (see details in Cappa et al. (2022c)), the 392 genotyped trees included a total of 52 parents, consisting of 47 mothers and five identified fathers. Among the 47 mothers, 34 belonged to the 40 OP families selected for SNP genotyping from the original 224 OP families that represented the range of height variation (Cappa et al. 2022c), while the remaining 13 originated from 35 potential forward-selected trees previously identified based on height BVs and included for sequencing. The number of trees per mother ranged from 1 to 11, with most of the underrepresented families originating from either the additional forward-selected trees or new families with an identified father. Overall, the final dataset comprised 47 OP families (including 348 trees) and six full-sib families (8 trees in total).

### Statistical analyses

#### Multitrait mixed models

We first fitted a pedigree-based additive (ABLUP) multitrait individual-tree mixed model (MTM) (ABLUP-MTM). Let $y_{ij}$ denote the phenotype of the tree *i* (*i* = 1, 2, …, *q*) for trait *j* (*j* = 1, 2, …, 9) (*i.e. j* indexes the set {HT, WGR, WD, RES, DECL, C13, LIMO, T_MONO, CAR}). If data are ordered such that trees are nested within traits, and letting $y^{\prime }=\;[y_{1}^{\prime },\;\ldots ,y_{9}^{\prime }]$, then the multitrait individual-tree model can be expressed in matrix notation as:


(1) $$\begin{array}{rl} \left[\begin{array}{c} y_{1}\\ \vdots \\ y_{9} \end{array}\right] & =\;\left[\begin{array}{ccc} X_{1} & \cdots & 0\\ \vdots & \ddots & \vdots \\ 0 & \cdots & X_{9} \end{array}\right]\left[\begin{array}{c} \beta_{1}\\ \vdots \\ \beta_{9} \end{array}\right]+\left[\begin{array}{ccc} Z_{a_{1}} & \cdots & 0\\ \vdots & \ddots & \vdots \\ 0 & \cdots & Z_{a_{9}} \end{array}\right]\left[\begin{array}{c} a_{1}\\ \vdots \\ a_{9} \end{array}\right]\\ & +\;\left[\begin{array}{c} e_{1}\\ \vdots \\ e_{9} \end{array}\right] \end{array}$$

where $\beta^{\prime }=\;[\beta_{1}^{\prime },\ldots ,\beta_{9}^{\prime }]$ is a vector of fixed effect of genetic groups and $a^{\prime }=\;[a_{1}^{\prime },\ldots ,\;a_{9}^{\prime }]$ is a random vector of additive genetic effects or BVs, which is assumed to be distributed as $a∼N(0,\Sigma_{a}⊗A)$, where $\Sigma_{a}$ is the additive genetic effects (co)variance matrix and A is the numerator relationship matrix derived from the pedigree (Henderson 1984) and containing the additive relationships among all trees. Finally, $e^{\prime }=\;[e_{1}^{\prime },\;\ldots ,e_{9}^{\prime }]$ is a vector of random errors distributed as $e∼N(0,R_{0}⊗I_{q})$, where $R_{0}$ is the error (co)variance matrix and $I_{q}$ is an identity matrix of order *q*. We assumed an unstructured (co)variance matrix for the genetic ($\Sigma_{a}$) and error ($R_{0}$) effects. The matrices $X_{j}$, and $Z_{a_{j}}$, relate the phenotype to the means of the genetic group effects in β, and the additive genetic effects in a. The apostrophe indicates the transpose operation whereas the symbol ⊗ stands for the Kronecker operator.

To be more explicit on the (co)variance structure of the model (1), note that $\Sigma_{a}$ and $R_{0}$ are matrices of order 9 × 9 with the following elements:


$$\Sigma_{a}=\;\left[\begin{array}{ccc} \sigma_{a_{1}}^{2} & \cdots & \sigma_{a_{1,9}}\\ \vdots & \ddots & \vdots \\ Sym & \cdots & \sigma_{a_{9}}^{2} \end{array}\right]\;\mathrm{and}\;R_{0}=\;\left[\begin{array}{ccc} \sigma_{e_{1}}^{2} & \cdots & \sigma_{e_{1,9}}\\ \vdots & \ddots & \vdots \\ Sym & \cdots & \sigma_{e_{9}}^{2} \end{array}\right]$$

where $\sigma_{a_{j}}^{2}$ and $\sigma_{e_{j}}^{2}$ are the additive and environmental variance parameters for trait *j*, respectively, and $\sigma_{a_{j,r}}$ and $\sigma_{e_{j,r}}$ represent the genetic and environmental covariance parameters between traits *j* and *r*.

A slight modification in the genetic (co)variance structure allows us to define the genomic multitrait model (GBLUP-MTM). In this model, the numerator relationship matrix (***A***-matrix) is replaced by the marker-based pairwise relationship ***G***-matrix. Following VanRaden (2008), the genomic relationship matrix (***G***-matrix) based on 25K SNPs was calculated as follows:


$$G=\frac{WW^{\prime }}{2\sum p_{k}(1-p_{k})}$$

where, W is the *q* × *m* (*q* = number of individuals, *m* = number of SNPs) rescaled genotype matrix defined by **M** - **P**, where **M** is the genotype matrix containing genotypes coded as 0, 1, and 2 according to the number of alternative alleles, and **P** is a vector of twice the allelic frequency of the *k*^th^ marker, $p_{k}$.

The distribution of realized pairwise relatedness coefficients estimated from the ***G***-matrix is shown in Supplementary Fig. 1. For the 392 genotyped trees, a total of 77,026 pairwise relationships were estimated (excluding two pairs corresponding to four full-sib trees from two families). Most comparisons (97.35%, *n* = 74,984) involved individuals classified as unrelated according to the pedigree, while 2.14% (*n* = 1,650) represented half-sib relationships. The heatmaps in Supplementary Fig. 2 further illustrate the family structure captured by both pedigree- and genomic-based relationships. While the ***A***-matrix reflects the expected relationships according to recorded parentage, the ***G***-matrix captures realized genomic similarity, including variation within and among families. These genomic and pedigree-based relationships were subsequently used to estimate variance components and each trait´s heritability in both the MTM and SEM models, using the ABLUP and GBLUP approaches.

Individual narrow-sense heritability for trait *j* (${\hat{h}}_{j}^{2}$) and genetic correlations between traits *j* and *r* (${\hat{r}}_{jr}$ ) were obtained as follows:


$${\hat{h}}_{j}^{2}=\frac{{\hat{\sigma }}_{a_{j}}^{2}}{{\hat{\sigma }}_{a_{j}}^{2}+{\hat{\sigma }}_{e_{j}}^{2}};\;{\hat{r}}_{jr}=\frac{{\hat{\sigma }}_{a_{j,r}}}{\sqrt{{\hat{\sigma }}_{a_{j,j}}^{2}\times {\hat{\sigma }}_{a_{r,r}}^{2}}}$$

where the point estimate of each (co)variance parameter was taken as the mean of its corresponding marginal posterior distribution.

The Bayesian approach, via Gibbs sampling, was used to estimate the parameters and predicted genomic BVs in model (1) by means of the MTM R-package (https://github.com/QuantGen/MTM). A single Gibbs chain of 510,000 iterations was sampled, where the first 10,000 iterations were discarded due to burn-in, and a thinning interval (thin) of two. Thus, 250,000 samples were used to compute summaries from the marginal posterior distribution. The posterior mean and 95% high posterior density interval (95% HPD) were then calculated using the coda R-package (Plummer et al. 2006). Convergence was monitored by visually inspecting the sample trace plots and posterior density plots, also using the coda R-package (Plummer et al. 2006).

#### Bayesian networks structural learning

As a preliminary step before fitting the SEM, we inferred the causal relationships among the nine traits using a Bayesian network (BN). To infer the causal relationships, the posterior mean of the predicted genomic BVs derived from the Bayesian GBLUP-MTM was used as input, as described in previous studies (Yu et al. 2019; Momen et al. 2021). Since BN learning algorithms assume independent response variables (Scutari and Denis 2021), a transformation via Cholesky decomposition was applied following Töpner et al. (2017) to remove dependencies among genotypes introduced by the genomic relationship matrix (***G***-matrix). Specifically, the ***G***-matrix was decomposed as ***G*** = ***LL′***, were ***L*** is a lower triangular matrix of order *q × q*. A transformation matrix $M=\;I_{9\times 9}⊗L$ was then defined, where $I_{9\times 9}$ is the identity matrix of order equal to the number of traits (9). The genomic BVs (a) were premultiplied by the inverse of this matrix ($M^{-1}$), yielding transformed genomic BVs $a^{*}=\;M^{-1}\;a$ that are independent across genotypes while retaining the genetic (co)variance structure among traits $\left(\Sigma_{a}⊗I_{q\times q}\right)$. This transformation satisfies the independence assumption required prior to BN learning.

In this study, four algorithms were applied to learn the underlying network structure at the genetic level: two score-based algorithms (Tabu search (Tabu) and Hill-Climbing (HC)), and two hybrid algorithms (Max–Min Hill-Climbing (MMHC) and 2-phase Restricted Maximization (RSMAX2)) (Scutari and Denis 2021). Score-based algorithms involve heuristic approaches to learning the BN structure, assigning a network score to each potential network, and maximizing it. Hybrid learning algorithms combine constraint-based and score-based methods to produce a reliable network structure and include two steps: restriction and maximization. These four algorithms were implemented using the R-package bnlearn v. 4.9.1 (Scutari and Denis 2021).

We employed a bootstrapping technique with 5,000 samples to assess the strength of edges and the uncertainty of their direction, following the method outlined by Scutari and Denis (2021). Subsequently, the 5,000 learning structures for each algorithm were averaged with a threshold for edge strength at 95% to produce a more robust network structure. Each of the four algorithms produced a competing BN, representing different underlying causal structures. These were compared using the Bayesian information criterion (BIC) and the Bayesian Gaussian equivalent score (BGe). Both scores were calculated for the entire network and for the network after removing each edge with a weight above the 95th percentile. In bnlearn, the BIC score is rescaled by −2, so a larger BIC indicates a preferred model.

#### Structural equation models

Based on the learned underlying structure inferred by the BN, two equation models (SEM were fitted (ABLUP-SEM and GBLUP-SEM). To describe the models, assume now that the data are ordered such that traits are nested within trees. Let $y_{i}^{\prime }=[y_{i1},\;\ldots ,y_{i9}]$ be the vector of records on the nine traits for tree *i*. Then, the SEM is defined in terms of the following equations:


(2) $$y_{i}=\Lambda y_{i}+X_{i}^{\prime }\beta^{*}+a_{i}^{*}+e_{i}^{*}$$

where $X_{i}^{\prime }\beta^{*}$, $a_{i}^{*}$, $e_{i}^{*}$ represent fixed effects, BVs, and error terms, and **Λ** is a 9 × 9 lower-triangular matrix of structural equation coefficients (*λ*), with off-diagonal elements taken from the network structure learned from the BN, and zeros along the main diagonal:


$$\Lambda =\left[\begin{array}{ccccccccc} 0 & 0 & 0 & 0 & 0 & 0 & 0 & 0 & 0\\ \lambda_{WGR\to LIMO} & 0 & 0 & 0 & 0 & 0 & 0 & 0 & 0\\ \lambda_{WGR\to HT} & \lambda_{LIMO\to HT} & 0 & 0 & 0 & 0 & 0 & 0 & 0\\ \lambda_{WGR\to CAR} & \lambda_{LIMO\to CAR} & \lambda_{HT\to CAR} & 0 & 0 & 0 & 0 & 0 & 0\\ \lambda_{WGR\to RES} & \lambda_{LIMO\to RES} & \lambda_{HT\to RES} & \lambda_{CAR\to RES} & 0 & 0 & 0 & 0 & 0\\ \lambda_{WGR\to WD} & \lambda_{LIMO\to WD} & \lambda_{HT\to WD} & \lambda_{CAR\to WD} & \lambda_{RES\to WD} & 0 & 0 & 0 & 0\\ \lambda_{WGR\to T_{MONO}} & \lambda_{LIMO\to T_{MONO}} & \lambda_{HT\to T_{MONO}} & \lambda_{CAR\to T_{MONO}} & \lambda_{RES\to T_{MONO}} & \lambda_{WD\to T_{MONO}} & 0 & 0 & 0\\ \lambda_{WGR\to DECL} & \lambda_{LIMO\to DECL} & \lambda_{HT\to DECL} & \lambda_{CAR\to DECL} & \lambda_{RES\to DECL} & \lambda_{WD\to DECL} & \lambda_{T_{MONO}\to DECL} & 0 & 0\\ \lambda_{WGR\to C13} & \lambda_{LIMO\to C13} & \lambda_{HT\to C13} & \lambda_{CAR\to C13} & \lambda_{RES\to C13} & \lambda_{WD\to C13} & \lambda_{T_{MONO}\to C13} & \lambda_{DECL\to C13} & 0 \end{array}\right]$$

Here, $\lambda_{\;j\to r}$ represents the rate of change of trait *j* with respect to trait *r*. If, based on the BN structure, trait *j* is not causally related to trait *r*, the corresponding *λ* The value was set to zero. Importantly, fixed effects, BVs, errors, and their corresponding (co)variance parameters generally differ from those in the MTM. However, if all elements of the **Λ** matrix are equal to 0, then the SEM model is equivalent to the MTM model (Oberpenning et al. 2023).

A Gibbs sampling chain with 510,000 samples was also generated to estimate the parameters and predicted genomic BVs from the SEM models, with the initial 10,000 samples discarded as burn-in, and a thinning interval of two iterations. We also used the R-package coda (Plummer et al. 2006) to compute summaries from the marginal posterior distribution and to assess the convergence. The MTM R-package was also used to fit the SEM models (https://github.com/QuantGen/MTM).

The goodness of fit for the pedigree- and genomic-based MTM and SEM models was assessed using the deviance information criterion (DIC), which combines a measure of total fit (the posterior expectation of the deviance, $\bar{D}(\theta_{M})$) with a penalty for model complexity (the effective number of parameters, *p*_D_) (Cappa and Cantet 2006). Similar to the Akaike information criterion (AIC), the DIC penalizes the additional parameters that improve the fit while promoting model parsimony. Therefore, models with lower DIC values are preferred, indicating better fit and a lower degree of complexity. The DIC was calculated as: DIC = $\bar{D}(\theta_{M})$ − *p*_D_.

Estimated BVs from the MTM (equation (1)) and SEM (equation (2)) obtained using both ABLUP and GBLUP approaches were compared across the nine studied traits. To visualize the relationship between BVs from both models, scatter plots were generated, displaying regression equations, Pearson correlations (Corr), and coefficients of determination (R^2^) to assess model agreement. Furthermore, ranking plots were created to compare the top 39 (∼10%) trees, with Spearman correlations calculated to evaluate the consistency of elite tree rankings across the two models. This analysis provides insight into the stability of selection rankings when applying different models (MTM and SEM) and approaches (ABLUP and GBLUP).

Finally, the predictive ability of the MTM and SEM models was evaluated using both ABLUP and GBLUP approaches through a ten-fold cross-validation analysis. In each fold, one subset of the data was used as the validation set, while the remaining nine subsets served as the training set. All trees with phenotypic data were included in the training population at least once across the 10 folds. Predictive ability was quantified as the Pearson correlation between the design-adjusted phenotypes of the validation subset and their corresponding predicted BVs from the respective models (ABLUP-MTM, GBLUP-MTM, ABLUP-SEM, and GBLUP-SEM).

## Results

### Bayesian Networks among genomic traits

Both the Bayesian Information Criterion (BIC) and Bayesian Gaussian equivalent (BGe) scoring methods consistently ranked the network structures, with score-based algorithms producing better fitting models than hybrid algorithms (Table 2). Among the score-based approaches, the Hill-Climbing (HC) algorithm generated the optimal network structure, achieving the best overall performance, as indicated by highest BIC and BGe scores. While the hybrid algorithms (MMHC and RSMAX2) showed slightly poorer fits, their BIC and BGe scores remained comparable.

**Table 2.** Bayesian information criterion (BIC) and Bayesian Gaussian equivalent (BGe) scores for the two score-based algorithms: TABU and HC (Hill-Climbing), and the two hybrid algorithms: MMHC (Max-Min Hill-Climbing) and RSMAX2 (2-phase Restricted Maximization).

Algorithms	BIC	BGe
TABU	−1,413.46	−1,398.98
HC	−1,405.31	−1,391.16
MMHC	−1,470.14	−1,457.87
RSMAX2	−1,470.14	−1,457.87

**Fig. 1.**
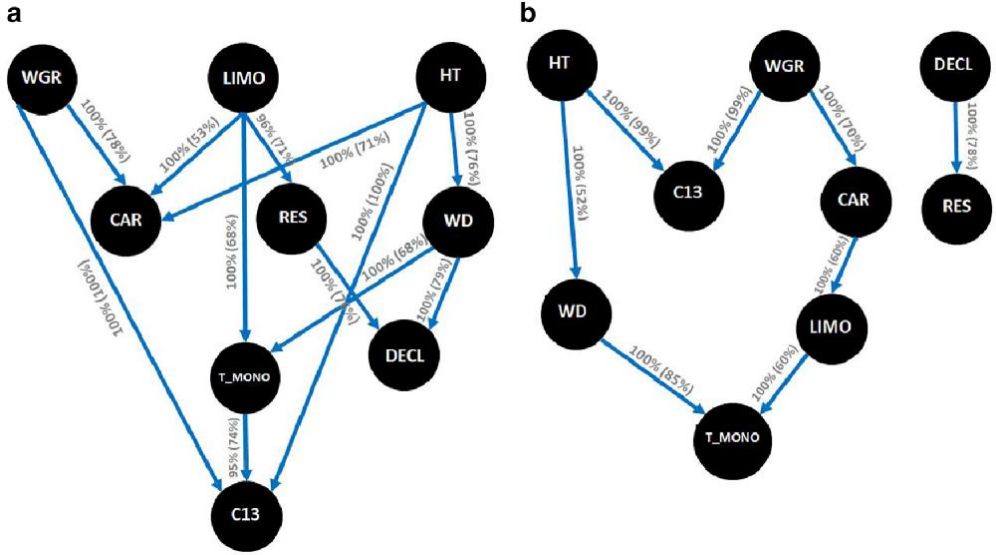
Bayesian networks based on the (a) score-based Hill-Climbing (HC) and (b) hybrid-based Max–Min Hill-Climbing (MMHC) algorithms. The inferred network structure was performed with 5,000 bootstrap samples. Labels on the edges indicate the strength and direction (parentheses), which measure the confidence of the directed edge. See text for trait abbreviations.

Figure 1 shows the top-performing network structures based on the highest BIC values, identified using the HC score-based algorithm and the MMHC hybrid algorithm. Interestingly, the network structure inferred by the RSMAX2 algorithm was identical to that of MMHC, so only the MMHC result is presented. Notably, the MMHC-generated network structure was a subset of the HC structure, differing only in the direction of the relationships between the traits DECL and RES (with HC showing RES → DECL and MMHC showing DECL → RES).

In the network produced by the HC algorithm (Fig. 1a), 10 out of the 12 edges were supported by 100% of the bootstrap samples in terms of their presence. However, the direction of the edges showed some variability in the level of confidence. For example, the dependency of C13 on WGR was strongly supported (100% of bootstrap samples), but there was less support to the direction of the path linking WGR to CAR (78% bootstrap samples). C13 connected to both HT and WGR, with both paths showing high confidence (100% bootstrap samples). T_MONO also played a mediating role between LIMO and C13, though its direction was less certain. Further, the HC algorithm identified several dependencies with directions moderately supported, such as the relationships between LIMO and RES (71%) and LIMO and CAR (53%). The path between HT and DECL was mediated by WD, but HT also had direct connections with both C13 and CAR, with moderate confidence (71 and 76% bootstrap support, respectively). Additionally, WD and RES had direct dependencies with DECL (79 and 76% bootstrap support, respectively), further highlighting the intricate interdependencies between these traits.

The deletion of some arcs in the HC algorithm resulted in notable increases in the BIC score, indicating that certain relationships are key to maintaining the optimal network structure. Specifically, removing the arcs WGR → C13, HT → C13, and LIMO → RES resulted in the largest BIC increases (Table 3), suggesting these pathways play a pivotal role in trait interconnections. The most influential arc identified by the HC algorithm was HT → C13, which also was the top-ranked arc in the MMHC algorithm, followed closely by WGR → CAR, CAR → LIMO, and WGR → C13. The consistent identification of these key pathways across different algorithms underscores the robustness of these relationships in the genomic network.

**Table 3.** Bayesian information criterion (BIC) scores for pairs of nodes reporting the change in the score caused by an arc removal relative to the entire network score for the two best algorithms (HC and MMHC). See text for algorithm trait abbreviations.

Algorithm	From	To	BIC
HC	HT	WD	3.09
	HT	C13	8.04
	HT	CAR	−0.05
	WGR	C13	10.02
	WGR	CAR	2.44
	WD	DECL	0.00
	WD	T_MONO	0.00
	RES	DECL	−3.12
	LIMO	RES	8.97
	LIMO	T_MONO	−1.12
	LIMO	CAR	−1.12
	T_MONO	C13	7.68
MMHC	HT	WD	7.78
	HT	C13	57.61
	WGR	C13	28.11
	WGR	CAR	32.57
	WD	T_MONO	24.63
	DECL	RES	10.75
	LIMO	T_MONO	10.75
	CAR	LIMO	32.57

In summary, the learned Bayesian networks (BN), particularly from the HC algorithm, provide valuable insights into the relationships between traits at the genetic level, with several pathways showing high support. The strong connections between HT, WGR, and C13, along with the involvement of other traits such as LIMO and RES, underscore the complex dependencies across these lodgepole pine traits.

### Model comparison: MTM vs. SEM

Table 4 provides the effective number of parameters (pD) and the DIC for the four models generated across the nine traits. For the MTM model, the effective number of parameters was 588.7 for ABLUP and 698.0 for GBLUP, while their corresponding DIC values were 6090.8 and 6630.5. In comparison, the SEM models showed higher effective parameters, with 1160.1 for ABLUP and 1189.7 for GBLUP, along with lower DIC values of 5783.7 and 6415.9, respectively. Notably, when comparing the DIC values between MTM and SEM for each model, the ABLUP model showed a decrease of 307.0 in DIC when transitioning from MTM to SEM, while GBLUP exhibited a reduction of 214.6. These lower DIC values in SEM suggest better model fit compared to MTM, with the reduction being more pronounced for ABLUP (5.04%) than for GBLUP (3.24%), highlighting the potential influence of causal effects among the nine key traits studied.

**Table 4.** Effective number of parameters (pD) and deviance information criterion (DIC) for the models analyzed. See text for model descriptions.

Model	ABLUP		GBLUP		Difference^a^	
	pD	DIC	pD	DIC	ABLUP	GBLUP
MTM	588.7	6,090.8	698.0	6,630.5	307.0	214.6
SEM	1,160.1	5,783.7	1,189.7	6,415.9		

^a^ DIC from MTM minus DIC from the respective SEM.

### Structural equation coefficients

The structure inferred from the BN among the nine genomic traits (Fig. 1) using the HC algorithm was used to define a set of structural equations. The structural coefficient matrix (**Λ**) describes the directional influence of one trait on another and is a key component of the SEM framework. This matrix quantifies how a change in one trait propagates through the network, influencing other traits. The resulting structural coefficient matrix is organized as follows:


$$\Lambda =\left[\begin{array}{ccccccccc} 0 & 0 & 0 & 0 & 0 & 0 & 0 & 0 & 0\\ 0 & 0 & 0 & 0 & 0 & 0 & 0 & 0 & 0\\ 0 & 0 & 0 & 0 & 0 & 0 & 0 & 0 & 0\\ \lambda_{WGR\to CAR} & \lambda_{LIMO\to CAR} & \lambda_{HT\to CAR} & 0 & 0 & 0 & 0 & 0 & 0\\ 0 & \lambda_{LIMO\to RES} & 0 & 0 & 0 & 0 & 0 & 0 & 0\\ 0 & 0 & \lambda_{HT\to WD} & 0 & 0 & 0 & 0 & 0 & 0\\ 0 & \lambda_{LIMO\to T_{MONO}} & 0 & 0 & 0 & \lambda_{WD\to T_{MONO}} & 0 & 0 & 0\\ 0 & 0 & 0 & 0 & \lambda_{RES\to DECL} & \lambda_{WD\to DECL} & 0 & 0 & 0\\ \lambda_{WGR\to C13} & 0 & \lambda_{HT\to C13} & 0 & 0 & 0 & \lambda_{T_{MONO}\to C13} & 0 & 0 \end{array}\right]$$

Table 5 provides the estimates of the 12 causal structural coefficients (λ) derived from the pedigree (ABLUP) and genomic-based (GBLUP) SEM models. These coefficients quantify the rate of change in *j* with respect to trait *r*, representing the direct genetic effect of one trait on another within the network structure.

**Table 5.** Posterior means of 12 causal structural coefficients (λ) derived from the two structural models studied (ABLUP and GBLUP) based on the network learned with the Hill-Climbing algorithm. See text for trait and model abbreviations.

Path	ABLUP	GBLUP
λ_HT→WD_	0.263	0.413
λ_HT→C13_	0.422	0.565
λ_HT→CAR_	−0.110	−0.254
λ_WGR→C13_	0.011	0.097
λ_WGR→CAR_	−0.048	−0.195
λ_WD→DECL_	0.117	0.096
λ_WD→T_MONO_	0.069	0.086
λ_RES→DECL_	−0.359	−0.509
λ_LIMO→RES_	0.111	0.205
λ_LIMO→T_MONO_	0.252	0.119
λ_LIMO→CAR_	0.194	0.368
λ_T_MONO→C13_	0.159	0.140

The estimates of these structural path coefficients varied across the models. Notably, the coefficients associated with HT showed significant effects on WD, C13, and CAR for both models, with generally higher values observed in the GBLUP-SEM model. For example, the path HT → WD and HT → C13 had structural coefficients of 0.263 and 0.422, respectively, under ABLUP, which increased to 0.413 and 0.565, respectively, under GBLUP. This indicates a stronger causal influence of HT on WD and C13.

The key paths included WGR → C13, where the coefficient increased from 0.011 in ABLUP to 0.097 in GBLUP; LIMO → CAR, which increased from 0.194 to 0.368; and LIMO → RES, which increased from 0.111 to 0.205. These changes highlight a stronger positive causal genetic effect of WGR on C13, of LIMO on CAR, and of LIMO on RES under the GBLUP framework compared to the ABLUP models. Additionally, the negative coefficients for HT → CAR and RES → DECL were also more pronounced under the GBLUP model (−0.254 and −0.509, respectively), indicating HT (and RES) exerts a strong negative causal effect on CAR (and DECL).

### Variance components and heritability estimates from the MTM and SEM

The posterior means and standard deviations of the variance components and heritability estimates for each trait studied are summarized in Table 6. Results are presented for both the MTM and SEM models, using two approaches: ABLUP and GBLUP.

**Table 6.** Posterior means and posterior standard deviations of variance components and heritability estimates from the multitrait (MTM) and structural equation models (SEM) for each trait studied. See text for trait abbreviations.

Model	Trait	ABLUP			GBLUP		
		Additive	Residual	Heritability	Additive	Residual	Heritability
MTM	HT	0.69 (0.14)	0.28 (0.11)	0.71 (0.12)	0.48 (0.13)	0.47 (0.11)	0.50 (0.12)
	WGR	0.58 (0.19)	0.49 (0.15)	0.54 (0.15)	0.68 (0.17)	0.40 (0.13)	0.63 (0.13)
	WD	0.62 (0.16)	0.43 (0.13)	0.59 (0.13)	0.60 (0.16)	0.44 (0.13)	0.58 (0.13)
	RES	0.43 (0.19)	0.63 (0.17)	0.40 (0.16)	0.30 (0.14)	0.75 (0.13)	0.28 (0.13)
	DECL	0.54 (0.23)	0.59 (0.19)	0.47 (0.18)	0.41 (0.20)	0.70 (0.18)	0.37 (0.16)
	C13	0.64 (0.15)	0.38 (0.12)	0.63 (0.13)	0.53 (0.15)	0.48 (0.12)	0.52 (0.13)
	LIMO	0.40 (0.13)	0.65 (0.12)	0.38 (0.11)	0.33 (0.12)	0.73 (0.11)	0.31 (0.10)
	T_MONO	0.31 (0.11)	0.72 (0.11)	0.30 (0.10)	0.29 (0.11)	0.73 (0.11)	0.28 (0.10)
	CAR	0.27 (0.12)	0.79 (0.11)	0.25 (0.10)	0.25 (0.11)	0.80 (0.11)	0.24 (0.10)
SEM	HT	0.59 (0.11)	0.35 (0.09)	0.63 (0.10)	0.51 (0.11)	0.44 (0.09)	0.53 (0.10)
	WGR	0.75 (0.18)	0.36 (0.12)	0.67 (0.12)	0.74 (0.15)	0.36 (0.10)	0.67 (0.10)
	WD	0.84 (0.16)	0.25 (0.11)	0.77 (0.11)	0.80 (0.16)	0.28 (0.12)	0.74 (0.12)
	RES	0.47 (0.18)	0.60 (0.16)	0.43 (0.15)	0.44 (0.16)	0.62 (0.14)	0.42 (0.13)
	DECL	0.43 (0.14)	0.68 (0.13)	0.38 (0.11)	0.48 (0.14)	0.62 (0.13)	0.44 (0.11)
	C13	0.85 (0.14)	0.21 (0.10)	0.80 (0.10)	0.72 (0.17)	0.34 (0.12)	0.68 (0.12)
	LIMO	0.54 (0.22)	0.54 (0.18)	0.49 (0.18)	0.38 (0.16)	0.67 (0.14)	0.36 (0.13)
	T_MONO	0.43 (0.21)	0.63 (0.18)	0.40 (0.18)	0.39 (0.15)	0.65 (0.13)	0.37 (0.13)
	CAR	0.61 (0.28)	0.54 (0.21)	0.52 (0.20)	0.49 (0.18)	0.63 (0.14)	0.43 (0.14)

Posterior means of variance components and heritability estimates exhibited some differences when comparing the two approaches across both models. In the MTM analysis, ABLUP heritability estimates ranged from 0.25 (±0.10) for CAR to 0.71 (±0.12) for HT, while GBLUP estimates showed a slightly lower range, with heritability values of 0.24 (±0.10) for CAR and a maximum of 0.63 (±0.13) for WGR. As highlighted by Beaulieu et al. (2022) in a metadata analysis of forest trees, these results suggest that ABLUP tends to produce higher heritability estimates compared to those obtained with GBLUP for most traits, particularly for HT and RES.

The differences between the two models (MTM and SEM) are somewhat anticipated because, in MTM, genetic effects encompass both direct and indirect influences on each trait, whereas in SEM, genetic effects reflect only the direct impacts acting specifically on each trait (*e.g.* Valente et al. (2013)). For both approaches, the SEM yielded lower posterior means of residual variances and higher posterior means of additive variances and heritability estimates for all traits compared to the MTM, except HT and DECL under the ABLUP model. For example, under ABLUP, the heritability estimates for CAR and WD increased from 0.25 (±0.10) and 0.59 (±0.13) in the MTM to 0.52 (±0.20) and 0.77 (±0.11), respectively, with the application of SEM. Similarly, for GBLUP, SEM estimates indicated higher heritability across all traits, with CAR showing the largest increase (79.17%), rising from 0.24 (±0.10) in MTM to 0.43 (±0.14) in SEM. In contrast, the upstream traits (WGR, LIMO, and HT; Fig. 1a) showed smaller differences between GBLUP-SEM and -MTM compared to the downstream traits (CAR, RES, WD, T_MONO, DECL, and C13; Fig. 1a) in terms of variance components and heritability estimates. On average, the differences for upstream traits were approximately 11% versus 43% for genetic variance, 8% versus 21% for residual variance, and 9% versus 39% for heritability estimates, respectively. This outcome aligns with expectations, as these upstream traits are not influenced by other traits in the Bayesian network (Fig. 1a).

### Trait correlations from the MTM and SEM

Posterior means of genomic-based correlations between traits obtained from the GBLUP-MTM and -SEM models are reported in Table 7 and Supplementary Fig. 3. Strong genetic correlations were observed between certain traits. For instance, a strong positive genetic correlation was found between HT and WD (0.47 for MTM and 0.32 for SEM), as well as between HT and C13 (0.49 for MTM and 0.46 for SEM). In contrast, a negative genetic correlation was observed between HT and CAR (−0.19 for MTM and −0.25 for SEM), implying a trade-off between height growth and carbon allocation. Similarly, a strong negative genetic correlation was found between RES and DECL (−0.48 for MTM and −0.49 for SEM), suggesting that increased drought resistance is associated with reduced growth compared to their early growth stages.

**Table 7.** Posterior mean of the genomic-based (GBLUP) correlations between traits from the multitrait (MTM) (upper triangular) and structural equation (SEM) (lower triangular) models for the nine traits studied. See text for trait abbreviations.

	HT	WGR	WD	RES	DECL	C13	LIMO	T_MONO	CAR
**HT**		0.01	0.47	−0.01	−0.01	0.49	0.11	0.24	−0.19
**WGR**	0.00		−0.07	0.00	0.16	0.29	−0.11	−0.14	−0.25
**WD**	0.32	0.00		−0.07	0.19	0.21	0.06	0.23	0.04
**RES**	0.00	0.00	0.00		−0.48	−0.12	0.17	−0.13	0.04
**DECL**	0.03	0.00	0.12	−0.49		0.18	0.08	−0.05	−0.02
**C13**	0.46	0.09	0.15	0.00	0.01		0.00	0.20	−0.11
**LIMO**	0.00	0.00	0.00	0.17	−0.08	0.02		0.04	0.17
**T_MONO**	0.04	0.00	0.11	0.03	0.00	0.13	0.20		0.05
**CAR**	−0.25	−0.22	−0.08	0.06	−0.03	−0.13	0.31	0.05	

The genomic correlations for the same trait pairs were highly consistent between the MTM and SEM models, as reflected by a Pearson correlation of 0.929 (Table 7, Supplementary Fig. 3). However, the MTM model generally produced higher correlation estimates across most paths compared to the SEM model, with exceptions observed for LIMO and T_MONO (0.04 vs. 0.20) and LIMO and CAR (0.17 vs. 0.31). While these estimates are not directly comparable due to differences in their definitions, it is evident that MTM genetic effects capture the overall genetic influence on each trait, encompassing both direct and indirect effects mediated through other phenotypic traits. In contrast, SEM genetic effects reflect only the direct genetic influence on each trait, excluding mediation by other traits in the model (Valente et al. 2013).

Additionally, the 12 causal relationships (λ) obtained from the GBLUP-SEM model (Table 5) showed a strong Pearson correlation (0.932) with the posterior mean of the genomic-based genetic correlations from the GBLUP-MTM model (Table 7 and Supplementary Fig. 4). This indicates that stronger genetic correlations between two traits are associated with larger causal relationships, independent of the genetic covariance between them (Varona and González-Recio 2023).

### Breeding value comparisons between MTM and SEM

**Fig. 2.**
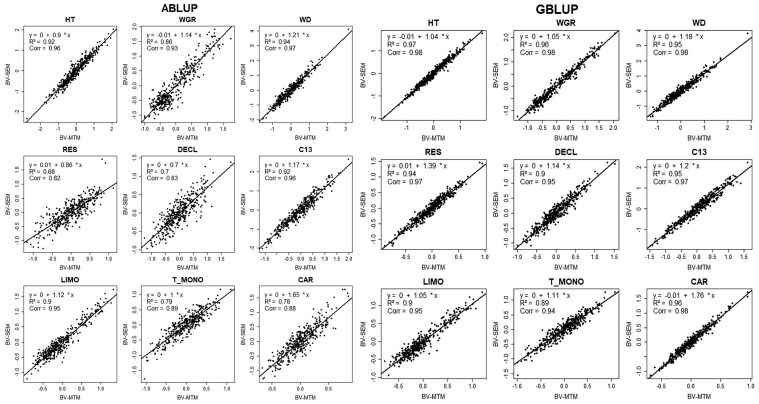
Scatter plot of the breeding values (BV) from the multitrait (MTM) (BV-MTM) and structural equation models (SEM) (BV-SEM) for each of the nine traits studied. Regression equation, coefficient of determination (R^2^), and Pearson correlations (Corr) are given in the upper left corner within each plot. The solid line indicates the regression line. See text for trait abbreviations.

The relationship between BVs estimated using the MTM and SEM models for two approaches: ABLUP and GBLUP, is shown in Fig. 2. Across all traits, high correlations are observed between the BVs from both models, with ABLUP correlations ranging from 0.82 to 0.97, and GBLUP correlations from 0.94 to 0.98. Traits such as HT and WD exhibit nearly perfect correlations between models, with correlations of 0.96 (ABLUP) and 0.98 (GBLUP) for HT and 0.97 (ABLUP) and 0.98 (GBLUP) for WD. Conversely, traits such as DECL and T_MONO show slightly lower correlations, with DECL having correlations of 0.83 (ABLUP) and 0.95 (GBLUP) and T_MONO correlations of 0.89 (ABLUP) and 0.94 (GBLUP). These findings indicate a strong overall agreement between the MTM and SEM models for most traits, though minor differences in the BV estimates are evident for traits such as DECL and T_MONO, where correlations are moderately lower.

**Fig. 3.**
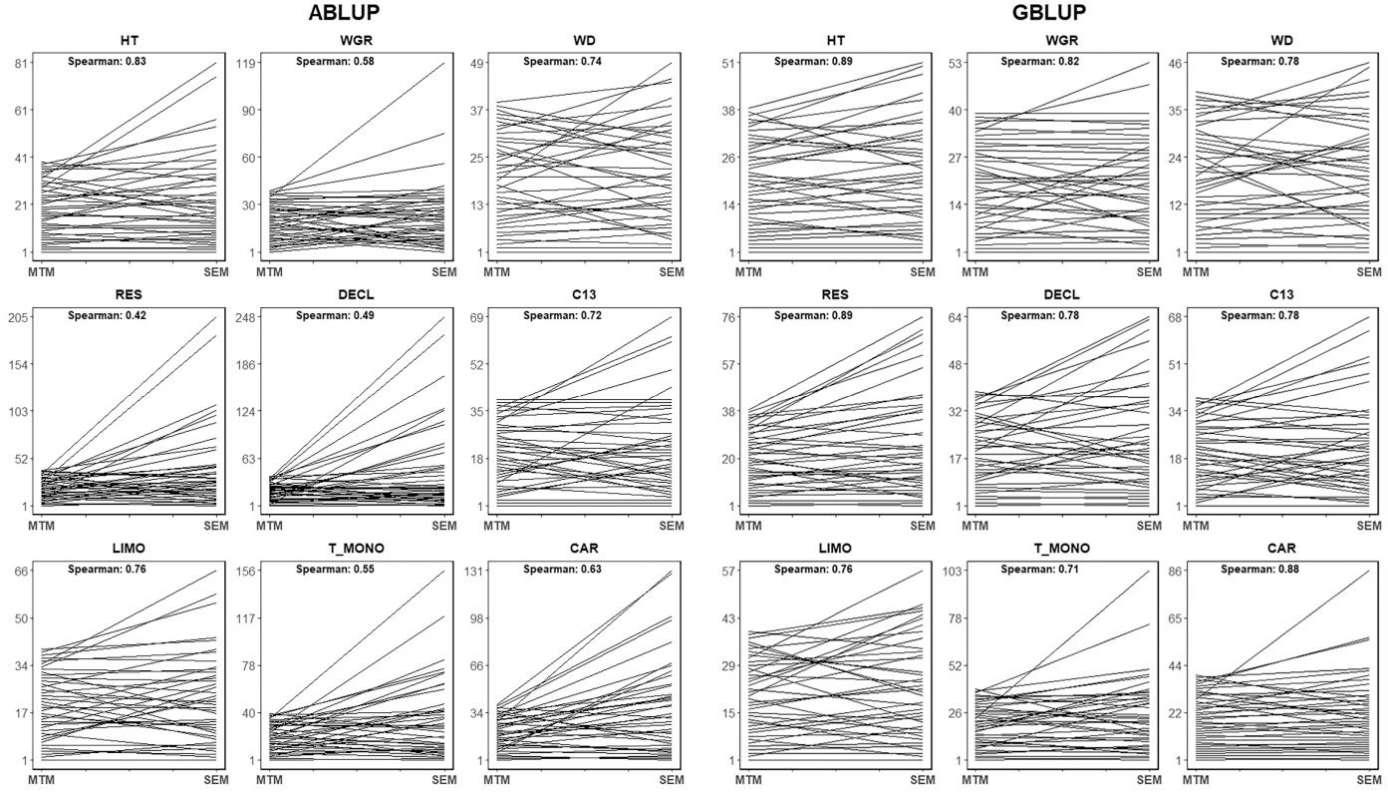
Ranking plots for the top 39 trees based on the breeding values of the multitrait (MTM) and structural equation models (SEM). The Spearman correlations are given in the upper left corner of each plot. See text for trait abbreviations.

Figure 3 presents ranking plots comparing the top 39 trees (10%) based on their BVs derived from the MTM and SEM models. The results indicate that GBLUP consistently showed higher Spearman correlations than ABLUP across the nine traits studied, with correlations ranging from 0.71 to 0.89 for GBLUP and 0.42 to 0.83 for ABLUP. However, the strength of these correlations varied across traits. High Spearman correlations were particularly notable for the upstream traits HT (0.83 for ABLUP and 0.89 for GBLUP), WD (0.74 for ABLUP and 0.78 for GBLUP), and LIMO (0.76 for both ABLUP and GBLUP), indicating strong consistency in elite trees ranking between the two models for these traits. In contrast, downstream traits such as DECL and T_MONO exhibited lower correlations, with DECL showing 0.49 (ABLUP) and 0.78 (GBLUP), and T_MONO showing 0.55 (ABLUP) and 0.71 (GBLUP). These lower correlations suggest that tree rankings for midstream or downstream traits are more sensitive to model choice, particularly for DECL and T_MONO. The variation in correlations implies that the SEM model, which accounts for causal relationships between traits, may affect the selection of top trees differently compared to the MTM model, especially for midstream or downstream traits that are more influenced by such causal effects.

### Predictive ability comparisons between MTM and SEM

**Fig. 4.**
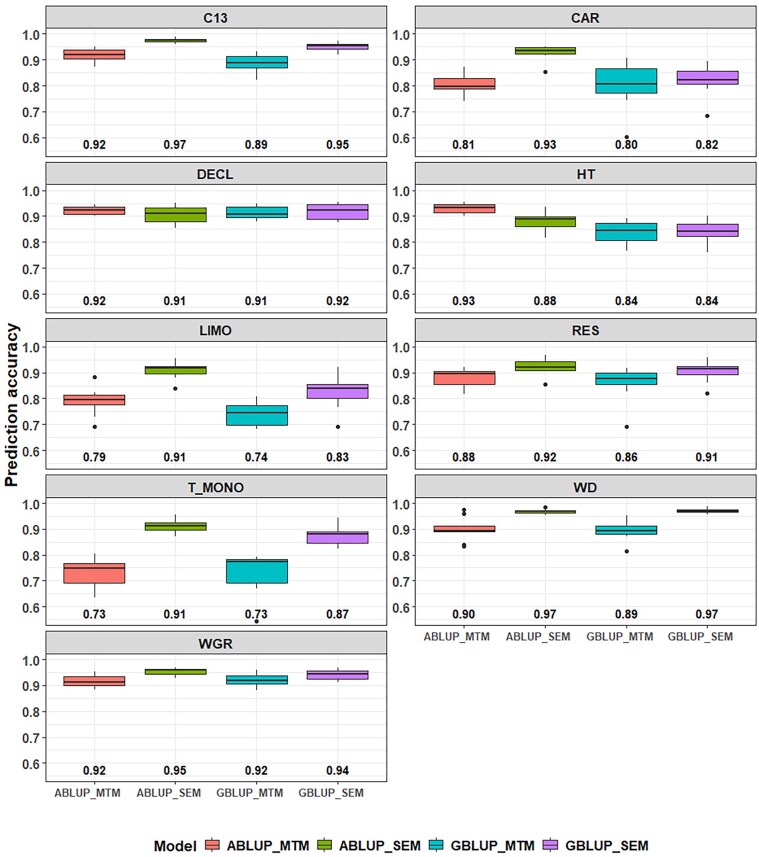
Box plots showing the predictive ability from the multitrait (MTM) and structural equation models (SEM) based on the pedigree (ABLUP) and genomic-based approaches (GBLUP) for each studied trait. Average predictive abilities are indicated below the box plots. See text for trait abbreviations.

The MTM and SEM models were also compared for both ABLUP and GBLUP approaches in terms of predictive ability (PA), estimated as the average Pearson correlation coefficient between the observed design-adjusted phenotypes and the predicted BVs for each trait (Fig. 4; Supplementary Table 1). Overall, the SEM models outperformed the MTM in both the ABLUP and GBLUP, with average gains across traits of 6.62 and 6.03%, respectively, except for HT under ABLUP. As expected, smaller differences between MTM and SEM were generally observed for upstream traits such as HT (0.01%) and WGR (2.22%), while more pronounced improvements were found for conditioned (midstream and downstream) traits such as T_MONO (19.71% for ABLUP and 19.14% for GBLUP), and C13 (5.69% for ABLUP and 7.18% for GBLUP).

## Discussion

Tree breeders should consider the genetic relationships between traits when making selection decisions, as improving one trait can affect the performance of another. Traditionally, MTM have been used to examine the shared genetic architecture underlying multiple traits. This study advances our understanding of trait interdependence in a lodgepole pine breeding program by exploring genetic interrelationships through both traditional MTM and the novel integration of BN with structural equation modeling (SEM). Employing both pedigree- and genomic-based approaches, this is, to the best of our knowledge, the first study to combine BN and SEM in forest tree breeding. By identifying a complex network and revealing critical connections between productivity, defense, and climate-adaptability traits, our study makes a significant advance in understanding complex trait relationships. The results demonstrate that, unlike traditional MTM, which attributes genetic correlations solely to pleiotropy or linkage disequilibrium between genes, the BN-SEM approach captures the causal and unidirectional influence of certain traits on other traits, shaping genetic covariances in ways not entirely explained by shared genetic effects. This provides a novel framework to improve selection and management strategies in forestry tree breeding.

### Genomic correlations from the MTM

The genetic correlations estimated from the GBLUP-MTM in this study (Table 7) reveal a complex interplay between the productivity, defense, and adaptability traits in the studied breeding population. Several noteworthy patterns emerged, carrying important implications for selection in the lodgepole pine breeding program.

A moderate to strong positive correlation was observed between height (HT) and both wood density (WD; *r_g_* = 0.47) and carbon isotope ratio (C13; *r_g_* = 0.49). This suggests that selecting for taller trees could inadvertently favour genotypes with higher WD and potentially improved water-use efficiency (WUE), as inferred from the C13. These relationships could be beneficial when both productivity and climate adaptation traits are targets for improvement in breeding programs. Many tree breeders employ HT (and the associated trait, volume) as a primary criterion for the selection of elite genotypes in breeding programs and, consequently, deployment, as this trait allows for the generation of maximal fiber production on a given site over a set rotation. In terms of wood quality traits, WD remains unequivocally the most important trait as it has a direct impact on the quality of products formed from the raw material. WD is known to directly influence the flexural properties (strength) of the wood, including modulus of elasticity (MOE) and modulus of rupture (MOR), as well as the machinability of the wood, rates of conversion, and the yield and fiber properties of pulp produced (Kang et al. 2004; Mansfield et al. 2007, 2016). Having a means to select for both HT and WD would be paramount for a tree breeding program. The relationship between growth rate and WD is expected to vary due to the complexity of WD as a trait, which is influenced by multiple factors. Among these, environmental conditions, such as location and site characteristics, appear to play a significant role in driving the differences observed within the same species (Zhang 1995). In addition to genetic and environmental influences, climate conditions also affect growth and wood traits across sites (Antony et al. 2014). Research on other conifers, including lodgepole pine, has reported a spectrum of genetic correlations between HT and WD, ranging from negative (Park et al. 2012), positive (Fries 1986; Wang et al. 1999; Cappa et al. 2022a), as well as weak to negligible correlations (Bouffier et al. 2009 ; Antony et al. 2014). In the same lodgepole pine population used in this study (Cappa et al. 2022a) found genetic correlations ranging from 0.13 to 0.60 were found between HT and WD across four sites, one of which was included in the current study.

The relationship between growth rate and C13 suggests that C13 could serve as a valuable proxy for selecting fast-growing genotypes with higher WUE. In the same lodgepole pine population, correlations between HT and C13 ranged from 0.08 to 0.55 (Cappa et al. 2022a). However, previous studies on field trials have not consistently shown a general trend between growth traits and WUE (Johnsen et al. 1999; Prasolova et al. 2000; Voltas et al. 2008; Marguerit et al. 2014). For instance, negative correlations between growth and C13 were observed in *Pinus halepensis* (Voltas et al. 2008), while positive correlations were found in *Pinus pinaster* (Marguerit et al. 2014).

The positive correlation observed between WD and T_MONO measured in phloem tissue (*r_g_* = 0.23), as well as with growth decline index (DECL, *r_g_* = 0.19), can be interpreted within the framework of plant defense strategies against biotic and abiotic stresses. These associations underscore the complex interplay between plant defense mechanisms and stress responses. Both increased production of monoterpenes and higher WD (associated with increased lignification) are well-established defensive strategies employed by trees under environmental stress (*e.g.* Bentz et al. (2017); Xie et al. (2018)). Similarly, the correlation with DECL suggests that, under stress conditions, trees may reallocate carbon resources away from growth and toward defense, resulting in increased investment in structural traits such as WD.

Similarly, WD and C13 also showed positive correlations (*r_g_* = 0.21), suggesting that improvements in WD are attributed to carbon assimilation traits. Comparable values were reported by (Cappa et al. 2022a) in the same lodgepole pine population (range: −0.12–0.34). Likewise, a positive genetic correlation between wood cellulose C13 and WD in three tropical tree species: *Tectona grandis*, *Acacia mangium,* and *Eucalyptus camaldulensis* has been reported (Halder et al. 2021). In *Fagus sylvatica*, Skomarkova et al. (2006) found that the relationship between WD and C13 has been shown to vary between dry and wet years across multiple sites, with a positive correlation between WD and isotope composition during dry years.

Although phenotypic correlations can often reflect nongenetic processes, similar to the findings of Sebastian-Azcona et al. (2025), who analyzed phenotypic relationships using the same lodgepole pine dataset but analyzed four progeny trials (Judy Creek, Swan Hills, Timeau, and Virginia Hills), we observed that high levels of WGR infection were associated at the genetic level with increased long-term decline (DECL) values (*r_g_* = 0.16). This association indicates a reduced drought response in affected trees and helps explain why trees affected by WGR appeared less capable of returning to predrought growth levels compared to healthy trees, as they likely struggled to fully take advantage of periods with sufficient water availability (Sebastian-Azcona et al. 2025).

In contrast, several trait pairs showed near-zero or very low correlations, such as WGR with RES (*r_g_* = −0.002) and HT with WGR (*r_g_* = 0.01). These results suggest that selecting for one trait in these pairs is unlikely to produce substantial indirect selection responses in the other. For example, the lack of genetic correlation between CAR and RES (*r_g_* = 0.04) or DECL (*r_g_* = −0.02) suggests that selection for CAR can occur independently without affecting traits related to drought response. This finding further corroborates earlier phenotypic-level results (Sebastian-Azcona et al. 2025), who used the same dataset but analyzed four progeny trials. Additionally, this outcome aligns with the findings of George et al. (2015), who reported weak correlations between WD parameters and drought response measures in *Abies* species, indicating that WD is an unreliable predictor of drought sensitivity. Overall, these results imply that xylem properties may not be the most critical traits for evaluating drought adaptation in conifers (Sebastian-Azcona et al. 2025).

Notably, a moderate to strong negative genetic correlation was found between RES and DECL (*r_g_* = −0.48). Therefore, selecting for trees with high drought resistance may result in individuals less capable of maintaining consistent growth over their lifetime, showing significant growth reductions compared to their early growth. Similarly, although less pronounced, WGR exhibited negative genetic correlations with several traits, including CAR (*r_g_* = −0.25) and monoterpene compounds (LIMO: *r_g_* = −0.11 and T_MONO: *r_g_* = −0.14). This suggests that higher levels of LIMO and total monoterpene concentration (T_MONO) may be associated with a reduction in WGR infection severity. Both LIMO and total monoterpene concentration, which have the highest relative composition in trees, are biologically significant for defense against mountain pine beetle (*Dendroctonus ponderosae*) (Erbilgin 2019). Meanwhile, selecting for reduced WGR infection severity may inadvertently promote higher carbon assimilation rates.

### MTM vs SEM: genetic parameters

Unlike MTM, SEM offers the significant advantage of distinguishing between genetic correlations and causal relationships among traits (*e.g.* Rosa et al. (2011); Varona and González-Recio (2023)). This distinction enables SEM to provide a more nuanced understanding of direct and correlated responses to selection, especially in scenarios involving nonlinear causal effects or when the causal effect opposes the additive genetic covariance (Varona and González-Recio 2023). Our findings revealed a strong correlation between the 12 causal structural coefficients (λ) estimated from the GBLUP-SEM model (Table 5) and the posterior mean of genomic-based genetic correlations from the GBLUP-MTM model (Table 7). Therefore, as stated by Varona and González-Recio (2023), these results suggest that most of the observed genetic correlations primarily arise from causal effects where one trait directly influences the other, rather than from pleiotropic effects of genes influencing both traits or linkage disequilibrium (LD) between different genes affecting each trait.

While the 12 causal structural coefficients (λ) demonstrated strong correlations with the genetic correlations (Corr = 0.932) (Supplementary Fig. 4), and the genetic correlations between both models (MTM and SEM) were generally high (Corr = 0.929), the posterior mean correlations estimated by the SEM models were consistently lower in magnitude than those obtained from the MTM, with the exception of the LIMO-T_MONO and LIMO-CAR pairs (Table 7). This aligns with findings from simulation studies that demonstrated that ignoring a recursive relationship leads to a significant overestimation of genetic correlations (Legarra and Robert-Granié 2006). Conversely, assuming a recursive relationship when none exists results in an underestimation of genetic correlations and regression coefficients deviating from zero. Similarly, simulations of a twelve-generation livestock population showed that, in contrast to SEM, MTM incorporates causal relationships into genetic correlations when they are present (Bonamy et al. 2024). The results found by Bonamy et al. (2024) could explain why the genomic correlations estimated in our study by MTM were higher than those from SEM in most cases, as MTM fails to disentangle causal relationships and instead absorbs them into the genetic correlation estimates. For example, in our study, the genetic correlation between HT and WD decreased from 0.47 to 0.32 (a reduction of 46.88%) under the SEM model. However, the λ between HT and WD was positive and strong (0.413), suggesting that a substantial portion of the observed association between HT and WD is mediated by direct genetic effects rather than pleiotropy or LD. In contrast, the genetic correlation between LIMO and CAR showed an increase of 45.16%, indicating that the SEM model accounted for additional shared genetic influences. The λ between these traits was also positive and strong (0.368), implying that much of this relationship can be attributed to direct causal effects. These results highlight how SEM provides a more nuanced interpretation of genetic correlations by disentangling direct genetic effects from indirect influences mediated through phenotypic pathways. Higher genetic correlations obtained from SEM compared to MTM have been previously documented in animal breeding studies (Mokhtari et al. 2018; Rahnein et al. 2023). However, other studies in animal breeding have reported very similar genetic correlations between the same traits across both models (Inoue et al. 2016; Oberpenning et al. 2023).

As expected, the increase in posterior means of variance components (and heritability estimates) from SEM to MTM was smaller for the unconditional (upstreaming) traits (WGR, LIMO, and HT) compared to the conditioned (midstream and downstream) traits (CAR, RES, WD, T_LIMO, DECL, and C13) (Table 6). Similar findings have been reported in animal breeding studies, such as for milk fatty acid traits (Bouwman et al. 2014), six meat quality traits in Wagyu beef (Inoue et al. 2016), and more recently, for five traits in a turkey population (Abdalla et al. 2021).

### Biological meaning of the inferred relationships

The relationships inferred from the causal structural coefficients (λ) offer biologically meaningful insights into the underlying mechanisms governing trait expression in interior lodgepole pine. Several of the relationships observed between growth, defense, and climate-adaptability traits reflect direction and potential drivers of key biological functions and expressions. For example, lodgepole pine HT showed a positive directional relationship with C13 (HT → C13 key arc; Table 3), and a negative relationship with CAR (HT → CAR arc; Table 5 and Fig. 1). These patterns align with research in other conifers showing that the direct effects of increased hydraulic resistance and gravitational effects with height positively affect tree-ring C13 while reducing stomatal conductance and increasing intrinsic water use efficiency (Ryan and Yoder 1997; Koch et al. 2004; Brienen et al. 2017; Vadeboncoeur et al. 2020). However, others have shown that positive correlations between height and C13 break down at warm test sites for lodgepole pine (Liu et al. 2023). As an estimate of integrated photosynthetic rate, intrinsic water use efficiency, and climate-adaptability, C13 appears to be a useful trait to identify genetic variation, as in other tree species, despite spatial, temporal, and environmental variation (Nelson and Johnsen 2008; Baldi and La Porta 2022).

In addition to these physiological links, the positive directional relationship of HT to WD (HT → WD arc; Table 5 and Fig. 1) highlights the potential to select for taller trees with greater WD in our study population and other lodgepole pine subpopulations. Although there is limited evidence of a positive directional relationship between HT and WD in lodgepole pine (Fries 1986; Wang et al. 1999), the association between growth traits and WD in our lodgepole pine populations is variable (Cappa et al. 2022a), reflecting the complexity of WD, a trait known to be influenced by multiple factors and many genes. For example, in addition to any genetic component, location along the stem, stand density, position within the stand (edge effect), soil type, nutrient status, and terrain aspect are all factors known to impact WD to varying degrees (Turvey and Smethurst 1985; Tomczak et al. 2020). The biomechanical requirement for strength to resist increased forces with height (Niklas 1993; Ducey 2012), particularly in the lower portion of the tree (*i.e.,* where WD was measured for this research) (Tomczak et al. 2020), supports our model suggesting a positive relationship between HT and WD (Fries 1986) in this lodgepole pine population. Furthermore, although the causal effect was very small (Table 5), greater WD contributed positively to growth decline under recurring drought (WD → DECL; Fig. 1), consistent with the trade-off between mechanical strength and hydraulic efficiency. These results align with known xylem-level mechanisms where higher-density wood often arises from narrower tracheids that reduce hydraulic conductivity but increase resistance to embolism formation under drought stress (Sperry et al. 2006; Bouche et al. 2014).

One of the more novel aspects of this study is the examination of associations among drought physiology, wood quality, and insect and disease resistance traits. Our results revealed a negative causal relationship from short-term resistance to drought (RES) to long-term growth decline (DECL) (RES → DECL arc; Table 5 and Fig. 1), consistent with the strong negative genetic correlation previously reported for this lodgepole pine dataset (*r_g_* = −0.48; Table 7 and Supplementary Fig. 3) and in line with biologically plausible patterns observed in both phenotypic and genetic relationships (Sebastian-Azcona et al. 2025). This prior knowledge supports the RES → DECL direction inferred by the HC algorithm rather than the opposite DECL → RES direction suggested by the MMHC algorithm, highlighting the value of incorporating prior biological knowledge to adjudicate among alternative directions when different BN learners produce conflicting results. Similar to other SEM applications in quantitative genetics (Bouwman et al. 2014; Peñagaricano et al. 2015; Mokhtari et al. 2025), such priors could be used to refine or biologically modify the inferred causal structure, ensuring that the final model remains consistent with well-established biological relationships among traits. Moreover, identifying traits known to be upstream in causal pathways and using them as conditioning variables can provide valuable insights into the direction and strength of causal relationships inferred by the BN (Rosa et al. 2011).

We also found a pattern where trees more susceptible and severely infected with WGR exhibited lower CAR (WGR → CAR arc; Table 5 and Fig. 1) and higher tree-ring C13 (WGR → C13 key arc; Table 3 and Fig. 1), supporting research that showed negative hydraulic effects of infection in well-watered lodgepole pine (Wolken et al. 2009), though higher water use efficiency, as inferred from C13 (Liu et al. 2023).

The allocation of carbon towards defense chemicals is reflected in the causal relationships inferred by the BN (Table 5 and Fig. 1). As expected, we found a strong positive correlation between LIMO and total monoterpenes (T_MONO), since LIMO is a component of total monoterpene concentration. Beyond this expected relationship, the relationships between defense and tree physiology traits revealed potential patterns of carbon allocation priorities. Specifically, higher defense concentrations of LIMO in the stem were positively associated with both CAR measured from needles (LIMO → CAR arc; Table 5 and Fig. 1) and inter-annual radial growth characteristics indicating drought resistance (RES) (LIMO → RES key arc; Table 3 and Fig. 1). This pattern suggests that trees with mountain pine beetle resistant traits, such as high concentrations of toxic LIMO (Ullah et al. 2021), can also have resistance to drought and subsequent growth decline after drought. (Liu et al. 2025) Reported that LIMO may function as a biostimulant by activating the plant antioxidant defense system, which was correlated in their experiment with biomass accumulation even under drought conditions. While our inferred causal structure supports the possibility that LIMO contributes to maintaining radial growth under drought, it also highlights the need for dedicated studies to elucidate the mechanisms underlying LIMO's role in drought resistance in conifers.

Furthermore, the positive but weak causal relationship between WD and T_MONO concentration (WD → T_MONO arc; Table 5 and Fig. 1) potentially indicates a growth-defense trade-off that has not been extensively explored but is supported by weak genetic correlations found between WD and terpene defenses in slash pine (Pinus *elliottii* Engelm. var. *elliottii*, (Ding et al. 2024)). Therefore, measuring multiple biotic resistance traits collectively with drought resistance physiology and dendrochronology traits provides an opportunity for data integration and enhancing our understanding of these complex relationships while revealing their strength, direction, and stability (Trugman et al. 2021; McDowell et al. 2022). Finally, the networks revealed instability in the causal relationships between LIMO and CAR (HC showing LIMO → CAR and MMHC showing CAR → LIMO, Table 5 and Fig. 1), underscoring the need for further research to clarify the regulation of biosynthetic response mechanisms linking tree defenses and physiology (Kaur et al. 2022; Li et al. 2024).

### MTM vs SEM: implications for tree breeding and selection

The interpretation of BVs for traits influenced by other traits (dependent traits) differs between SEM and MTM (Varona and González-Recio 2023). In SEM, BVs reflect the direct genetic effects on dependent traits, adjusted for the influence of independent traits, whereas in MTM, they capture the total additive genetic effects, encompassing both direct and indirect influences. For example, as similarly discussed by Varona and González-Recio (2023), the BVs from the GBLUP-SEM model for LIMO incorporate a positive causal effect on CAR (λ_LIMO→CAR_ = 0.368), despite a small genetic correlation of 0.17, indicating no pleiotropy or LD between the genes affecting LIMO and CAR. In contrast, BVs from the MTM model include genetic effects related to both LIMO and CAR, while SEM BVs for LIMO only account for its genetic effects without the influence of CAR.

The practical breeding impact of different models using the ABLUP and GBLUP approaches was assessed via correlations between BV predictions (Fig. 2), and the rank of the top 39 (10%) trees (Fig. 3). Our results showed that for traits such as LIMO and HT, which are causal root nodes in the network (*i.e.* not influenced by other traits; Fig. 1a), the BVs of the top-ranking 10% of trees selected using the MTM and SEM models exhibited strong Spearman correlations (Fig. 3). This suggests high consistency in selection for these upstream traits, regardless of the model employed. In contrast, for traits positioned midstream or downstream in the causal network, such as T_MONO and DECL (Fig. 1a), the Spearman correlations between BVs from the MTM and SEM models were considerably lower, indicating greater variation in selection outcomes depending on the model applied (Fig. 3). These findings align with the recent work of Bonamy et al. (2024), who found that animals selected based on traits at the top of the causal network remained consistent across generations, irrespective of the simulated scenario, *i.e.* whether assuming no genetic correlations across traits or when traits were both causally and genetically linked. However, they reported noticeable differences in selection outcomes for upstream traits when selection pressure was applied to midstream or downstream traits, reinforcing the idea that selection at lower network levels can have broader implications across the trait network. Moreover, these same authors found that in situations where a group of traits is causally connected but not genetically correlated, employing an SEM to estimate BVs allows for the genetic enhancement of a target trait without producing an indirect selection response on traits that precede it in the causal pathway. This finding is also significant for forest tree breeding programs. In our study, for example, using the BN (Fig. 1a), we observed that HT and DECL are causally connected through WD but have a very low (near-zero) genetic correlation (*r_g_* = 0.03), as shown in the genetic correlation matrix (Table 7). Using an SEM in this case allowed for selection on DECL without significant unintended consequences on the upstream HT trait. This contrasts with the MTM approaches, where BVs for traits upstream in the causal network, such as HT, would indirectly affect the selection of downstream traits such as C13, due to their positive genetic correlations (*r_g_* = 0.49).

The advantage of SEM in these scenarios is further demonstrated by Bonamy et al. (2024), who noted that selection on the target trait (in their case, trait 4) produced a significantly greater response under MTM than SEM. These results are because BVs from MTM incorporate the genetic correlations between traits upstream in the network, leading to indirect selection on correlated traits (such as HT and CAR in our case, where HT has a strong correlation with WD, *r_g_* = 0.47). As a result, MTM BV-based selection could enhance downstream traits indirectly, while SEM provides more precise selection for the target trait by minimizing such correlated responses.

In line with these observations, understanding the different sources of genetic correlations among traits can also help enhance the genetic progress of breeding programs. Our findings further demonstrate that incorporating causal relationships among traits improves the accuracy of estimated BVs (Fig. 4), results consistent with previous findings reported in animal breeding (Mokhtari et al. 2018; Razmkabir et al. 2020; Abdalla et al. 2021). As suggested by Rosa et al. (2011), when traits are causally connected, relying solely on MTM-based analyses may lead to suboptimal selection decisions, slower genetic progress than expected, and the neglect of important indirect synergies among traits.

Valente et al. (2013) highlighted that MTM-based predictions retain all relevant information under standard settings and are necessary for breeding purposes, even when traits are causally linked, as MTM accounts for both direct and indirect genetic effects. Consequently, BVs derived from MTM encompass the complete genetic information needed for effective predictions, particularly when the focus is on overall genetic effects. However, SEM explicitly models the interrelationships among traits, providing deeper insights into the biological processes driving trait variation and enabling more precise predictions of intervention outcomes. This uncovering of the interrelationship among traits can be especially valuable in forest tree breeding.

## Conclusion

In this study, we explored genomic relationship patterns among nine complex traits related to productivity, defense, and climate adaptability by integrating phenotypic and genomic data using BN and SEM. The BN-inferred structures and SEM provided richer insights than simple trait-association values from MTM. By distinguishing causal relationships between traits and differentiating them from genetic correlations, this integrated approach provides valuable information to guide more precise breeding decisions. SEM in particular enables more realistic modeling of biological relationships between traits, leading to improved parameter estimation and predicted BVs. While promising and still underutilized in forest tree breeding, BN-SEM provides a powerful framework to disentangle direct and indirect genetic effects, offering novel insights into the genetic causal architecture of key traits, particularly for midstream or downstream traits, and enabling more precise selection while minimizing unintended correlated responses. Our results revealed 12 causal relationships identified by the HC algorithm, with three key pathways: HT → C13, WGR → C13, and LIMO → RES demonstrating robustness within the genomic network. Incorporating these relationships into SEM resulted in lower residual variances, higher additive variances, and increased heritability estimates for both ABLUP and GBLUP approaches compared to MTM. Although genetic correlations from SEM closely aligned with MTM estimates (Corr = 0.929), SEM correlations were slightly lower, reflecting its inherent ability to isolate causal and genetic effects. BV correlations between SEM and MTM models showed strong consistency across traits, though traits like DECL (>0.83) and RES (>0.82) exhibited slightly lower correlations, with SEM providing more biologically supported results through recursive model estimation. Using SEM was shown to contribute to a more reliable prediction of selection candidates in both pedigree- and genomic-based approaches, thereby supporting more effective and informative breeding decisions. Moving forward, the use of SEM models, particularly those based on genomic data, holds great promise for improving the accuracy of BV predictions and enhancing the efficiency of selection programs in forest genetics.

## Supplementary Material

jkaf308_Supplementary_Data

## Data Availability

Genotyping-by-sequencing (GBS) raw reads used in this study have been deposited in NCBI SRA BioProject - PRJNA715165: https://www.ncbi.nlm.nih.gov/bioproject/715165. Information on the lodgepole pine trials, including pedigree and phenotypic data used and analysed during the current study, is available in the GitHub repository: https://github.com/RESFOR/quantitative_genetics_R/blob/main/Lodgepole_Pine_Phenotypic_and_Pedigree_DATA.txt. Supplemental material available at G3 online.
