# Supplementary material for: Bayesian networks and structural equation models reveal genetic causal relationships between productivity, defense, and climate-adaptability traits in interior lodgepole pine
Source: G3 (Bethesda). 2025 Dec 24;16(3):jkaf308. doi: 10.1093/g3journal/jkaf308 (PMC12958823; doi:10.1093/g3journal/jkaf308)

**Figure S1. Distribution of realized pairwise relationship coefficients (from the genomic relationship matrix, G) for genotyped lodgepole pine trees in the Timeau population, including trees belonging to the same family (expected value = 0.25), different families (expected value = 0), and self-relationships (expected value = 1).** Expected relationship values (0, 0.25, and 1) correspond to the pedigree-based expected means for unrelated, half-sib, and self comparisons, respectively.


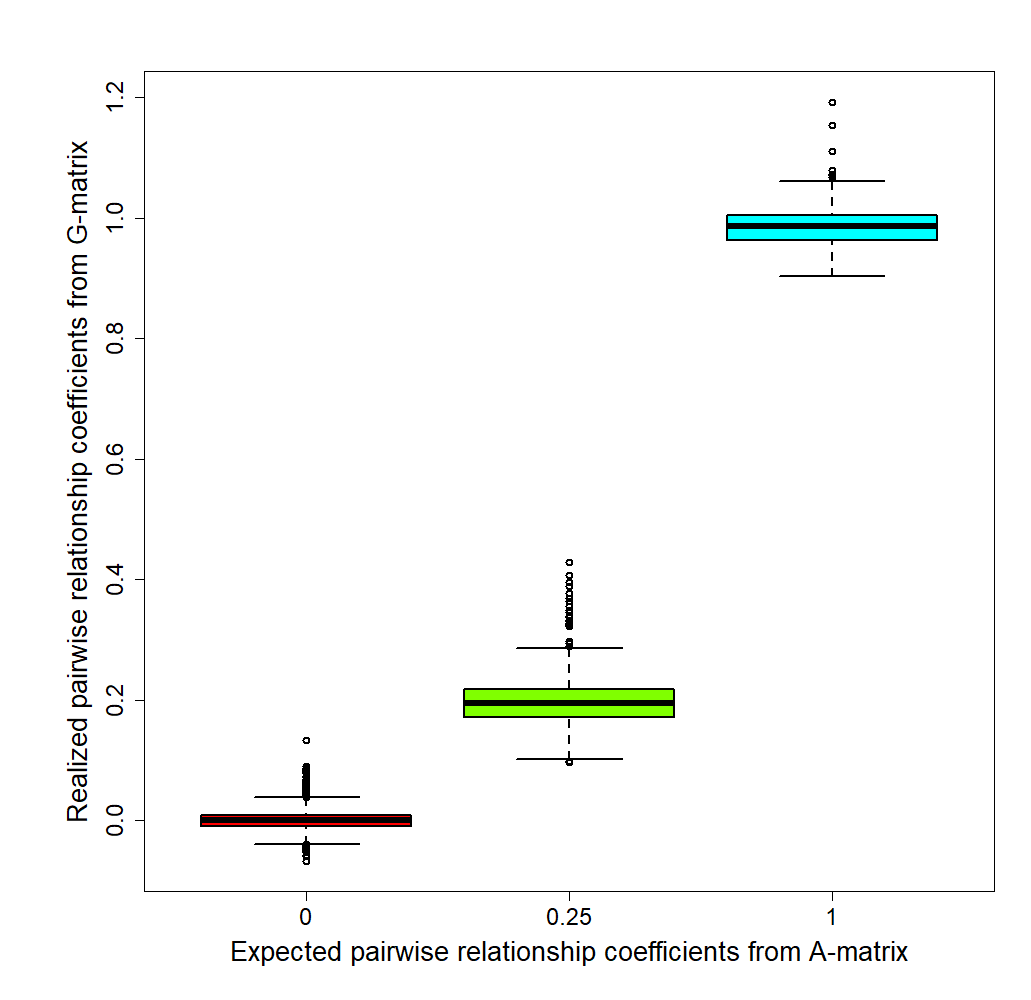

Supplement: jkaf308_Supplementary_Data [file jkaf308_supplementary_data.zip › Figure_S1._G3-2025-406403.docx]
