# Supplementary material for: Bayesian networks and structural equation models reveal genetic causal relationships between productivity, defense, and climate-adaptability traits in interior lodgepole pine
Source: G3 (Bethesda). 2025 Dec 24;16(3):jkaf308. doi: 10.1093/g3journal/jkaf308 (PMC12958823; doi:10.1093/g3journal/jkaf308)
Supplement: jkaf308_Supplementary_Data [file jkaf308_supplementary_data.zip › Figure_S2._G3-2025-406403.docx]

**Figure S2. Heatmaps of pairwise relationship coefficients among 392 lodgepole pine individuals. Left: Genomic-based relationships (*G*-matrix). Right: Pedigree-based relationships (*A*-matrix).** Individuals are ordered by mother, highlighting family structure along the diagonal. Off-diagonal values represent shared pedigree or genomic similarity between individuals from different families. Light blue squares along the diagonal indicate family clusters, while warmer colors correspond to higher relatedness.

| ***A***-matrix | ***G***-matrix |
| --- | --- |
| 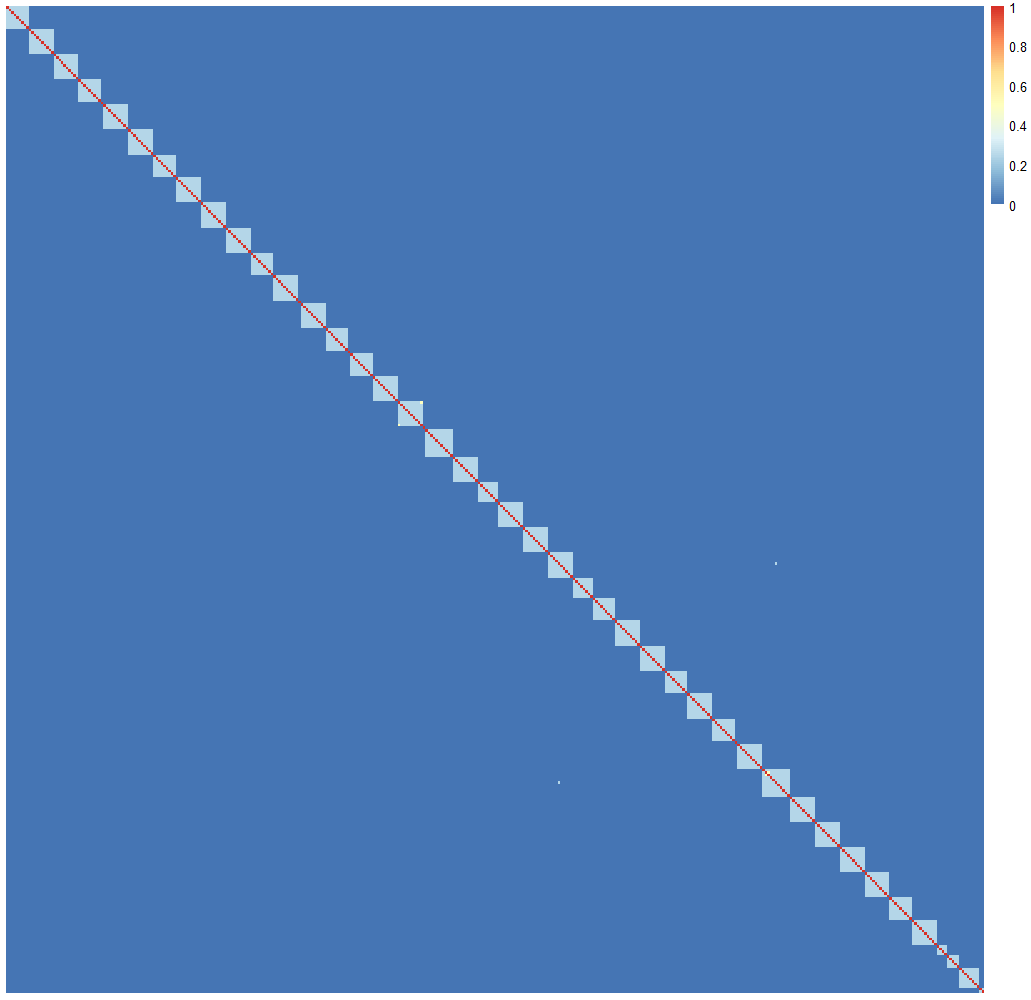 | 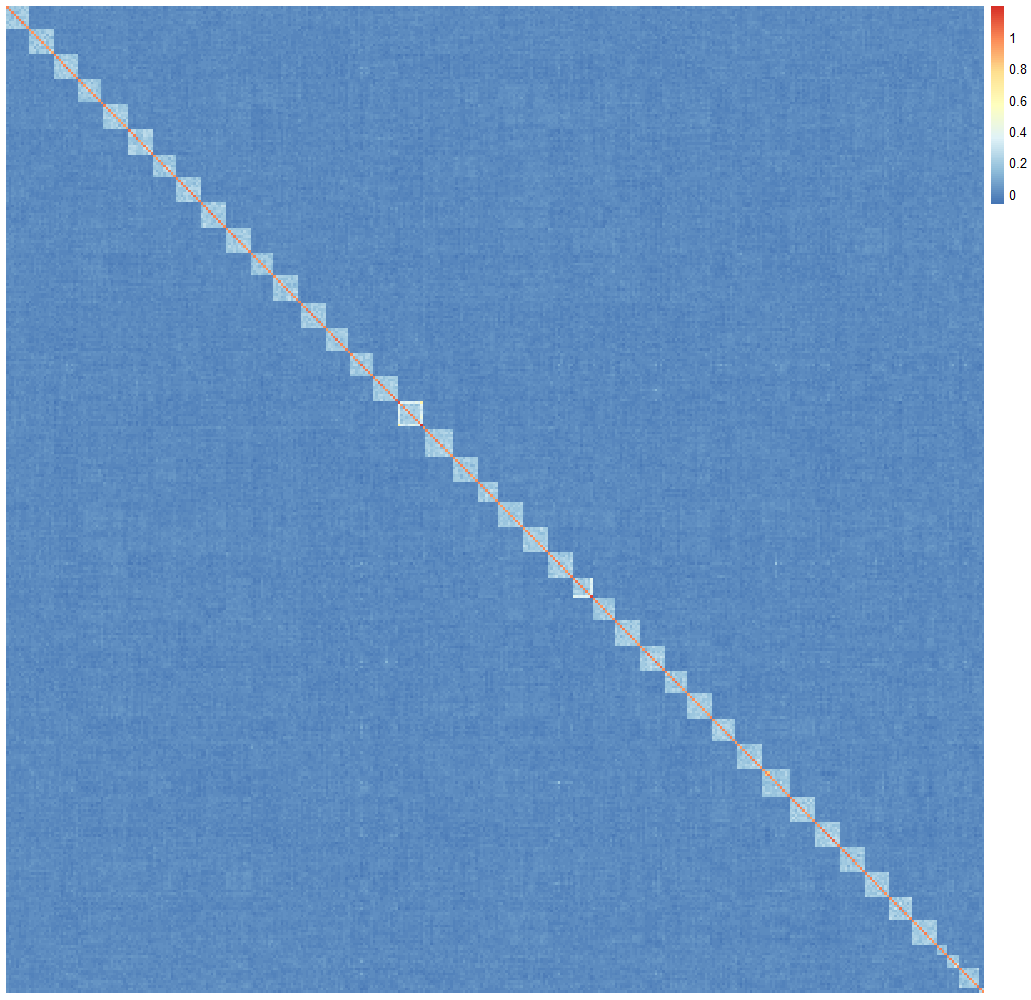 |
