# Supplementary material for: Bayesian networks and structural equation models reveal genetic causal relationships between productivity, defense, and climate-adaptability traits in interior lodgepole pine
Source: G3 (Bethesda). 2025 Dec 24;16(3):jkaf308. doi: 10.1093/g3journal/jkaf308 (PMC12958823; doi:10.1093/g3journal/jkaf308)
Supplement: jkaf308_Supplementary_Data [file jkaf308_supplementary_data.zip › Figure_S3._G3-2025-406403.docx]

**Figure S3. Posterior mean of the genomic-based correlations from the (A) GBLUP multi-trait (MTM) and (B) structural equation (SEM) models for the nine traits studied.** The colours and values correspond to the correlation between each pair of traits. The red and blue squares reflect negative and positive correlations, respectively. See text for trait abbreviations.

| **A** | 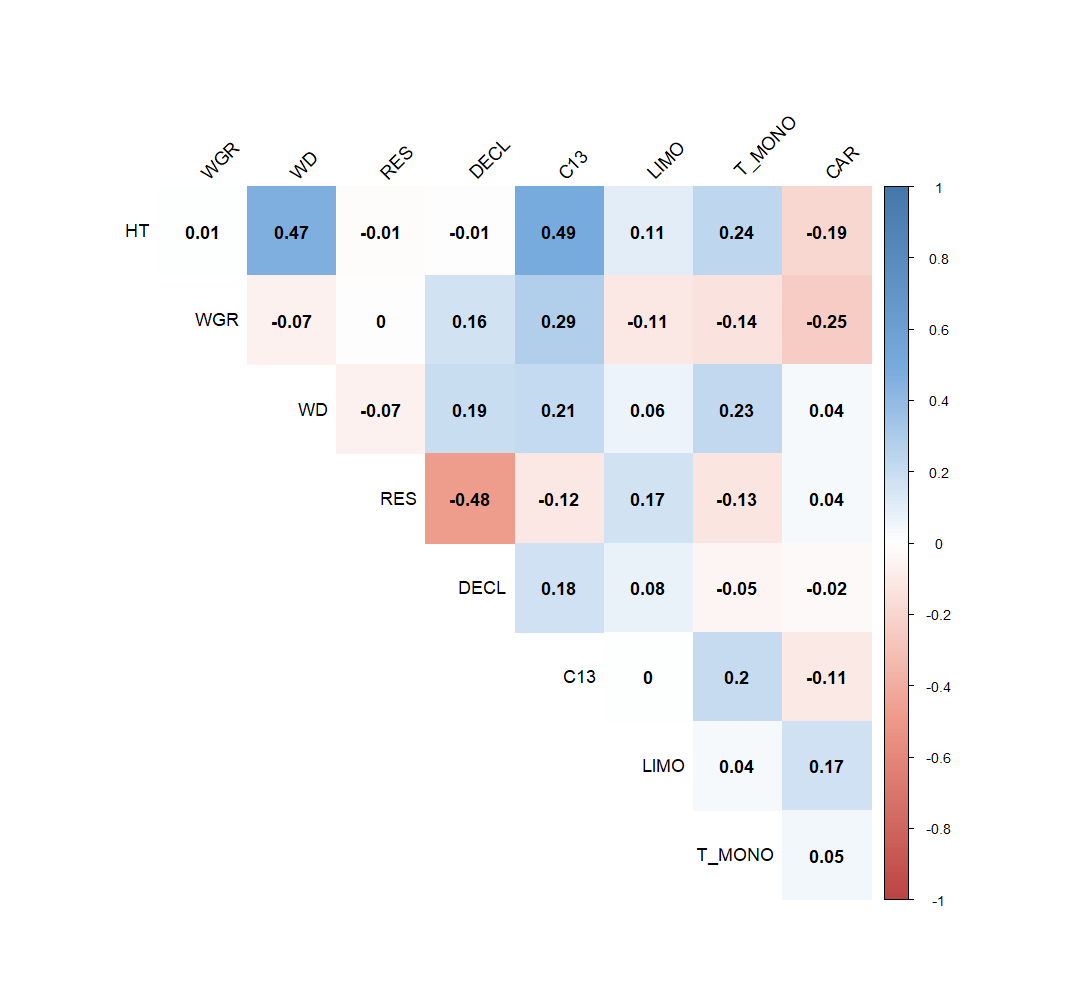 |
| --- | --- |
| **B** | 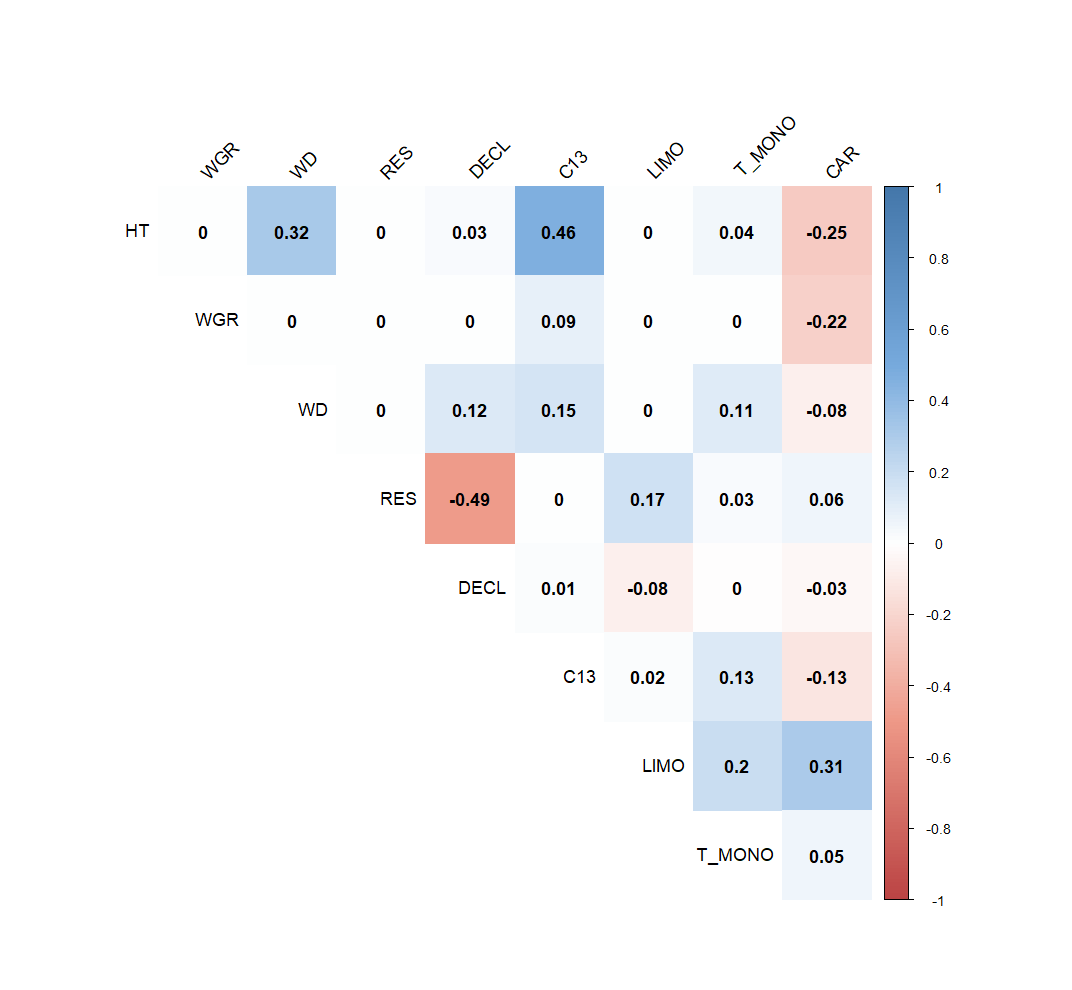 |
