# Supplementary material for: Bayesian networks and structural equation models reveal genetic causal relationships between productivity, defense, and climate-adaptability traits in interior lodgepole pine
Source: G3 (Bethesda). 2025 Dec 24;16(3):jkaf308. doi: 10.1093/g3journal/jkaf308 (PMC12958823; doi:10.1093/g3journal/jkaf308)

**Figure S4. Scatter plot of the posterior means of the 12 causal relationships (λ) estimated from the GBLUP-SEM model versus the posterior means of the genomic-based genetic correlations obtained from the GBLUP-MTM model.** The Pearson correlation coefficient between both sets of estimates is provided.


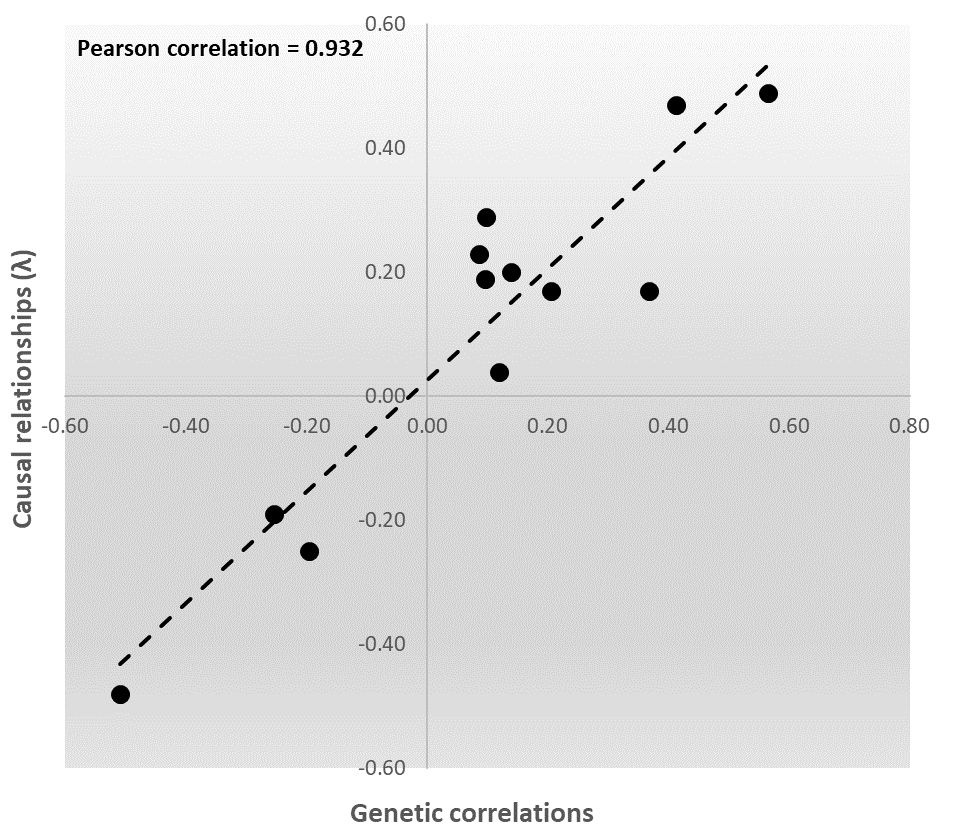

Supplement: jkaf308_Supplementary_Data [file jkaf308_supplementary_data.zip › Figure_S4._G3-2025-406403.docx]
