# Supplementary material for: Bayesian networks and structural equation models reveal genetic causal relationships between productivity, defense, and climate-adaptability traits in interior lodgepole pine
Source: G3 (Bethesda). 2025 Dec 24;16(3):jkaf308. doi: 10.1093/g3journal/jkaf308 (PMC12958823; doi:10.1093/g3journal/jkaf308)
Supplement: jkaf308_Supplementary_Data [file jkaf308_supplementary_data.zip › Table_S1._G3-2025-406403.docx]

**Table S1. Average predictive ability (and standard deviation) from the multi-trait (MTM) and for structural equation models (SEM) based on the pedigree- (ABLUP) and genomic-based approaches (GBLUP) for each studied trait**. See text for trait abbreviations.

| **Trait** | **ABLUP** | | **GBLUP** | | |
| --- | --- | --- | --- | --- | --- |
|  | **MTM** | **SEM** | | **MTM** | **SEM** |
| **HT** | 0.93 (0.02) | 0.88 (0.04) | | 0.84 (0.04) | 0.84 (0.04) |
| **WGR** | 0.92 (0.02) | 0.95 (0.01) | | 0.92 (0.02) | 0.94 (0.02) |
| **WD** | 0.90 (0.04) | 0.97 (0.01) | | 0.89 (0.04) | 0.97 (0.01) |
| **RES** | 0.88 (0.04) | 0.92 (0.03) | | 0.86 (0.07) | 0.91 (0.04) |
| **DECL** | 0.92 (0.02) | 0.91 (0.03) | | 0.91 (0.02) | 0.92 (0.03) |
| **C13** | 0.92 (0.02) | 0.97 (0.01) | | 0.89 (0.04) | 0.95 (0.02) |
| **LIMO** | 0.79 (0.05) | 0.91 (0.04) | | 0.74 (0.05) | 0.83 (0.07) |
| **T_MONO** | 0.73 (0.06) | 0.91 (0.02) | | 0.73 (0.08) | 0.87 (0.04) |
| **CAR** | 0.81 (0.04) | 0.93 (0.03) | | 0.80 (0.09) | 0.82 (0.06) |
